# Equilibrium distributions of simple biochemical reaction systems for time-scale separation in stochastic reaction networks

**DOI:** 10.1098/rsif.2014.0054

**Published:** 2014-08-06

**Authors:** Bence Mélykúti, João P. Hespanha, Mustafa Khammash

**Affiliations:** 1 Department of Mathematical Stochastics, University of Freiburg, Eckerstraße 1, 79104 Freiburg, Germany; 2 Centre for Biological Systems Analysis (ZBSA), University of Freiburg, Habsburgerstraße 49, 79104 Freiburg, Germany; 3 Electrical and Computer Engineering, University of California, Harold Frank Hall, Santa Barbara, CA 93106-9560, USA; 4 Department of Biosystems Science and Engineering, ETH Zürich, Mattenstrasse 26, 4058 Basel, Switzerland

**Keywords:** stochastic reaction kinetics, time-scale separation, quasi-steady-state assumption, gene regulation, dimer transcription factor, analytic combinatorics

## Abstract

Many biochemical reaction networks are inherently multiscale in time and in the counts of participating molecular species. A standard technique to treat different time scales in the stochastic kinetics framework is averaging or quasi-steady-state analysis: it is assumed that the fast dynamics reaches its equilibrium (stationary) distribution on a time scale where the slowly varying molecular counts are unlikely to have changed. We derive analytic equilibrium distributions for various simple biochemical systems, such as enzymatic reactions and gene regulation models. These can be directly inserted into simulations of the slow time-scale dynamics. They also provide insight into the stimulus–response of these systems. An important model for which we derive the analytic equilibrium distribution is the binding of dimer transcription factors (TFs) that first have to form from monomers. This gene regulation mechanism is compared to the cases of the binding of simple monomer TFs to one gene or to multiple copies of a gene, and to the cases of the cooperative binding of two or multiple TFs to a gene. The results apply equally to ligands binding to enzyme molecules.

## 1. Introduction

The model reduction of multiscale biochemical systems is a step of fundamental importance towards the system-level understanding of gene regulation or of various signalling pathways. Often reaction pathways consist of many intermediate reactive species that themselves are not of interest in a high-level interpretation of dynamic behaviour. Stripping these off to create a skeleton model is the goal of model reduction.

The field is heavily influenced by the chemical engineering and control engineering literature. The first methods were developed for deterministic ordinary differential equation (ODE) models, often for applications in the petrochemical industry. For the reaction rate ODE, there are many chemical systems where an approximating lower dimensional ODE model can be derived by time-scale separation techniques, such as the *quasi-steady-state* (QSS) *assumption* (QSSA) and *quasi*-*equilibrium assumption* (e.g. Michaelis–Menten kinetics or the bacteriophage *λ* lysis–lysogeny pathway [1]), or other methods (e.g. balanced truncation, lumping of variables). Subsequently, such techniques found extensive use in the mathematical biology community.

Recently, stochastic analogues of methods for deterministic systems are being developed. The QSS approximation and the quasi-equilibrium approximation are such examples, which have already been applied to the standard discrete Markov jump process model [2–5], and directly to its stochastic differential equation (SDE) approximation, the chemical Langevin equation (CLE) [6,7]. Applications to speed up Gillespie's stochastic simulation algorithm (SSA) [8] gave the *slow-scale SSA* [9] and the *nested SSA* [10]. The differences between the two approaches were discussed by both sets of authors [11,12]. Further contributions by the Petzold group include [13,14], and an analysis of the legitimacy of the Michaelis–Menten approximation in the stochastic setting [15]. Dong *et al*. [16] proposed reading out a reduced reaction system for the stochastic model from the model reduction of the corresponding deterministic reaction rate equation.

A sophisticated, general, flexible, although laborious technique to reduce multiscale stochastic models of chemical reaction systems was developed by Ball *et al*. [17]. Their starting point, the discrete-state Markov jump process, was written with a stochastic equation formalism in terms of independent Poisson processes for each reaction channel. In order to exploit the natural separation of the abundances of reacting species and time scales, scaling constants were introduced for each molecular species, each reaction channel and time. With the fine control of all these scaling parameters, the authors could approximate the different variables and reaction channels with diffusion approximations (SDEs) or continuous deterministic processes (integral equations), depending on the inherent scaling properties of the system. However, they could not provide rules for the appropriate choice of scaling constants, which is a great hindrance to the application of this model reduction technique. This shortcoming was addressed in [18].

Model reduction by the averaging method for the CLE was discussed in [7]. The authors compute the quasi-stationary distribution of the fast variables of the system given the slow ones from the Fokker–Planck equation (Kolmogorov forwards equation). The slow variables for the reaction intensity functions in the CLE are computed as averages with respect to this distribution.

In the derivation of the CLE [19], passing to a continuous limit in each variable uniformly is an approach of limited validity. Kang *et al.* [20] addressed the question of how to most accurately represent fluctuations in stochastic models of multiscale chemical systems with normally distributed noise. While this is very similar to the work of Sotiropoulos *et al.* [7], one important difference is that here the starting model is the discrete-state Markov process, as opposed to the CLE, as in [7]. The approach by Kang *et al.* is more careful and it does not need a restrictive assumption of Sotiropoulos and co-workers, the linear independence of the fast and the slow stoichiometric subspaces.

Pahlajani *et al*. [21] used singular perturbation on another SDE model of chemical kinetics, the linear noise approximation of Van Kampen, in order to eliminate chemical species with fast characteristic time scales and to obtain reduced models.

Berglund & Gentz [22,23] studied singular perturbation for general SDEs with both slow and fast dynamics. In the case when the corresponding deterministic system admits an asymptotically stable slow manifold, they prove that the paths of the SDE are concentrated close to this manifold and give upper and lower bounds for the probability of leaving this neighbourhood.

The focus of this study is equilibrium distributions (also known as stationary or invariant distributions) in biochemical reaction networks. Equilibrium distributions receive less attention in molecular systems biology, analogously to the steady states of ODE models, since interesting dynamic behaviour happens outside of equilibrium, by definition. Equilibrium distributions have been observed to provide less information than transient measurements for network identification in metabolic networks [24] and for parameter identification in gene expression [25]. An exception to this trend has been metabolic control analysis, which is interested in optimizing flows in steady state [26], but its modelling framework tends to be the deterministic reaction rate equation. This is partially due to historical reasons, partially due to metabolites being present in a cell in greater quantities than constituents of the gene expression machinery, hence intrinsic noise is a lesser concern.

This study emphasizes that accurately describing the equilibrium distribution is an indispensable step in the endeavour to understand multiscale reaction systems. It is this observation that justifies our interest in the subject.

First, our basic notions and Markov process modelling framework will be presented. Afterwards, the connection between the fast and the slow time scales by averaging or QSSA will be briefly discussed. These will be followed by the main contribution of this study, an extensive collection of biochemical systems for which the equilibrium distributions can be analytically computed. Additionally, this will lead to a comparison of different gene regulatory mechanisms.

For applications, it is most beneficial to create a catalogue of systems where one can look up equilibrium distributions. The classification will not be driven by the biological role of the biochemical systems. Instead, we ask what the Markov process state space topologies are for which we can express the equilibrium distribution in a meaningful way. We present these Markov processes if we can equip them with an interpretation as a biologically significant reaction network.

In the literature, the approaches of Levine & Hwa [24] and Bintu *et al*. [27] are most similar to ours. Levine & Hwa [24] focused on typical motifs of metabolic pathways: linear and unidirectional pathways, and extensions with additional structure, such as reversible reactions, dilution, negative feedback from end-product to the first reaction, or branching either in a diverging or in a converging fashion. Bintu *et al*. [27] catalogued a wide range of transcription regulatory systems: single and dual activators and repressors, systems of helper and regulatory molecules. Using concepts of thermodynamics, they expressed equilibrium distributions in terms of the Boltzmann distribution. While their method is readily applicable to configurations to which ours is not yet, our purely kinetic approach has the advantage that it uses reaction rates and rate constants (as opposed to binding energies), and therefore it can be integrated with dynamical simulation algorithms easily.

We do not review here the wide variety of results for two-stage gene expression models (the transcription of DNA to mRNA followed by translation to protein), only mention that important contributions include [28–31]. Williams and co-workers [32] derived the joint equilibrium distribution of protein species processed or degraded by a shared enzyme (or by multiple copies thereof).

Our principal technical contribution is a calculation for §4.6 that applies complex analysis (saddle-point integration) to product-form equilibrium distributions (§4.5). This technique is relevant in situations where a marginal of a product-form equilibrium distribution must be computed in a system with conservation.

## 2. Preliminaries

We assume that a number of chemical substances 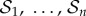 undergo reactions through reaction channels 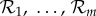 inside a cell, modelled as a well-stirred solution of fixed volume and temperature. It is implicit in the assumption that an exhaustive list of the reactive molecular species and their interactions is available. The reactions are of the form
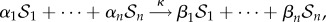
where 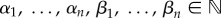 (non-negative integers) and not all zero, and 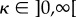 is a *reaction rate constant*, whose role will become clear shortly. If the above reaction is 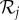 and we let *α* = (*α*_1_, …, *α*_*n*_)^T^ and *β* = (*β*_1_, … ,*β*_*n*_)^T^, then the *stoichiometric matrix* 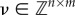 of the reaction system has *j*th column *ν*_*j*_ = *β* − *α*.

The state of the reaction system is described by the state vector 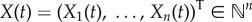 whose *i*th coordinate *X_i_*(*t*) gives the count of molecules 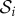 in the solution at time *t*. Associated to each reaction 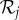, there is a so-called *propensity* (or *intensity*) *function* 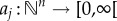. For the purposes of this study, we restrict ourselves to at most bimolecular (at most second-order) reactions (∑*_i_*α*_i_* ≤ 2). We also stipulate that the propensity functions satisfy the *law of mass action*. Under these conditions, the left-hand side of any reaction is one of four types: 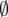, 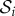, 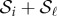 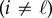, 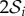. The corresponding propensity functions are 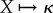, 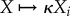, 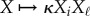, 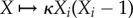, respectively, with the appropriate reaction rate constants *κ*.

With these tools, a continuous-time Markov process can be defined on the state space 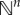. This is the standard stochastic process model of chemical reaction kinetics, which even today still does not seem to have an established name but is known by its Kolmogorov forwards equation, the *chemical master equation* [33], or by its standard simulation method, the *SSA* [8]. The transition rule of the Markov process is that in any state *X*, for each 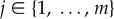, transitions corresponding to reaction 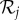 occur at rate *a_j_*(*X*). A transition by reaction 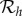 changes state *X* to *X* + *ν*_*h*_. Equivalently, if at time *t*_0_ the state is *X*(*t*_0_), then to each reaction channel 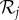 there is an associated independent, exponentially distributed random waiting time with parameter *a_j_*(*X*(*t*_0_)). The shortest of these waiting times specifies the reaction that occurs, say *R_h_* with waiting time *τ*. Then the state of the Markov process is *X*(*t*_0_) for times in [*t*_0_, *t*_0_ + *τ*[, and *X*(*t*_0_ + *τ*) = *X*(*t*_0_) + *ν*_*h*_. The update step is repeated with fresh independent exponential waiting times, first with parameters *a_j_*(*X*(*t*_0_ + *τ*)) for each *j*, then with the propensity functions evaluated at subsequent states of the process.

The time evolution of the probability mass function of this process is given by the chemical master equation [33]. For each element of the state space 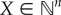, the probability of the process being in *X* at time *t*, conditioned on that it was in 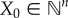 at some time *t*_0_ ≤ *t*, is
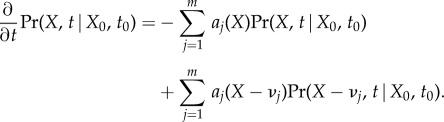


We simplify the notation by dropping the dependence on the initial state. Instead of writing out the ODE for each state *X*, we can fix an enumeration of all states (*X*^1^, *X*^2^,*…*) and write the equation in matrix form. For the vector of probabilities *P*(*t*) = (Pr(*X*^1^, *t*), Pr(*X*^2^, *t*),*…*),2.1
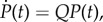
where the *rate matrix Q* satisfies
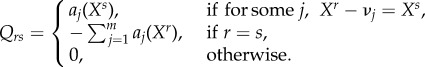


A distribution 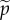 is an *equilibrium* (or *stationary* or *invariant*) *distribution*, if 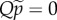.

Although the complete state space 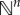 is countably infinite, most of our examples have finite state spaces. (The only exception has state space 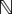, which is, however, naturally enumerated.) The reason is that often conservation relations partition the state space into stoichiometric compatibility classes. Conservation laws are in correspondence with left nullvectors of *ν*, because they are nothing but linear combinations of different species' counts that are preserved in all reactions. In such a case, the state space of the Markov process consists of disjoint classes that are pairwise inaccessible from one another. (Feinberg discussed this in the case of ODEs [34]. The case of discrete jumps on a discrete-state space is more involved because congruence classes—remainders—come into play.) Stationarity of a distribution can be determined on these classes separately. Any convex combination (mixture) of the equilibrium distributions of the separate classes is an equilibrium for the whole state space. In many cases, such as in most of our examples, these classes are finite, thus the probability vectors and rate matrices we study are finite-sized as well.

In applications where the set of accessible states is infinite, by omitting infinitely many states of low probability, a truncation of the set of accessible states to a finite set is possible at a cost of a small error in the probability vector with an explicit error bound [35].

We remark that finding an equilibrium distribution is the same as finding a right nullvector to *Q*. In linear algebra, this is an easy problem in the finite case that can be solved with Gaussian elimination. However, our aim is to exploit the structure of the rate matrix in order to find informative formulae for these distributions, and for this reason we do not content ourselves with algorithmic linear algebraic solutions.

## 3. Connecting fast and slow time scales

In this section, it is shown how the ability to explicitly compute equilibrium distributions is used in the study of biochemical reaction systems with multiple time scales. The approximation of multiscale reaction systems with reduced models is not a completely solved problem. At present, there is no solution to questions like what the most accurate representation of an *n*-variable stochastic reaction system with 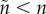 variables is, or how many variables are needed to achieve a required accuracy.

Unsurprisingly, many early results are inferred from (and in some cases only apply to) toy models and are based on heuristics. We highlight two challenges to an exhaustive solution. First, many studies treat systems with two time scales, whereas applications often have more than two time scales. Second, it is sometimes not individual species, but their linear combinations that have clearly separated fast and slow dynamics.

A case in point is this example [10]
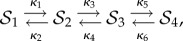
a network we equip with 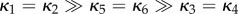. It is immediate that the network possesses three time scales. With an initial state of (1000, 0, 0, 0)^T^, the dynamics is clear: initially 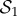 molecules transform into 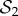 molecules, and this mixture of 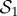 and 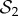 loses mass that turns into 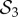, and then into a mixture of 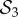 and 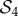. Each species is a participant in reactions that belong to the fast or the medium time scales. Still, the linear combinations 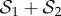 and 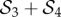 only change on the slow time scale. It is manifest in this example that important dynamics might happen on a slow time scale even though none of the individual species is a slowly changing species. Therefore, it is necessary to consider linear combinations of species in addition to individual species when disentangling multiscale dynamics.

An intuitive exposition of the QSS analysis method is found in [3]. However, owing to the cited challenges, the method in its completeness is mathematically technical and is thoroughly detailed in [18, §§4.2 and 6]. Here, we outline only the basic principle, without elaborating the conditions under which the QSSA holds. We also ignore the above presented challenges.

In the ideal setting, the chemical system has two well-separated time scales. The rapidly changing variables are assumed to behave like a Markov process under fixed values of the slowly changing variables, to have stable, ergodic dynamics, and consequently, to converge to a unique equilibrium distribution. The time-scale separation should be so great that the slow variables can be assumed to stay largely unchanged in the time it takes the fast variables to reach their unique equilibrium.

The effect of the rapidly changing species on the slow time scale is through this equilibrium distribution. That is, in the slow time scale, one uses the expectation of the slow-scale propensities under the equilibrium distribution of the fast-changing variables conditioned on the actual value of the slow variables.

Consider an example of regulated gene expression where a single gene is regulated by a rapidly binding and unbinding transcription factor (TF). Of interest is the dynamics of mRNA transcription intensity 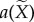, as a function of the reduced state vector of slow variables 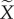, to be inserted into the model of the slow time scale. According to the preceding, 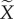 might include constant or slowly varying linear combinations of fast species. Let 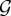 denote the gene in the free form and 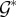 in the gene–TF complex form. It is assumed that the total number of genes is one, and that the total number of TFs (one of which might be bound) is constant on the fast time scale. Let Pr_QSS_ denote the QSS distribution of the gene and TF system, so that 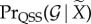 is the probability under the QSS distribution of the gene being free given the slowly changing variables, and 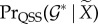 is the probability of the gene being in the complex form. Let the transcription propensity for the free (unbound) form *a*_u_ and for the gene–TF complex (bound) form *a*_b_ be given. Then, under the QSSA, according to the law of total expectation, the effective mRNA expression rate is
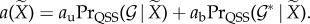
Whether the TF is an activator or a repressor is irrelevant, its effect is encoded in the relative magnitudes of *a*_u_ and *a*_b_.

Here, the complete state vector has three coordinates in addition to the mRNA count and possibly other variables: it contains the number of free genes, the number of gene–TF complexes and the number of free TFs. These three variables are fast. However, owing to the conservation relation for the total number of genes and owing to the slowly varying nature of the total number of TFs (assumed constant on the fast scale), only one of these three is independent and two are dependent variables on the fast scale. On the slow scale, this independent variable is assumed to be in equilibrium. Hence, the slow variable 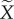 is dependent only on the total TF number, it does not differentiate between free and bound TFs. While the average transcription intensity 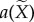 is dependent on the total TF number, *a*_u_ and *a*_b_ are not, and the dependence is purely through the equilibrium binding probability.

We note that in contrast to the QSSA (the approach we adopt), the quasi-equilibrium assumption stipulates that each pair of fast reversible reactions is balanced, that is, they are in detailed balance (cf. §4.1.1), a requisite of thermodynamic equilibrium. The quasi-equilibrium assumption is more restrictive than the QSSA, since formally, it often imposes conditions on reaction rate constants, and effectively, it rules out probability and mass flows on cycles of the state space. The fast dynamics converge to an equilibrium distribution under general conditions, but there is often no reason to expect that detailed balance will hold in the equilibrium.

To apply the QSSA in stochastic simulation, the starting point can be [9,10]. While Cao *et al*. [9] carefully demonstrated how to check that the time-scale separation assumption holds, understandably no general method was given as to how to compute the expectation of the slow-scale propensities. The authors proposed to compute this, when nothing else succeeds, by approximations, e.g. with a normal distribution or by moment closure. E *et al*. [10] proposed to simulate independent realizations of the fast dynamics and to compute the effective reaction propensities as a numerical average. The latter is a general but computationally more intensive method, and unfortunately it has no chance of being exact. A third method [36] entails the simulation of the fast dynamics until equilibrium is approximately reached (to be determined by a test for approximate detailed balance), and this is followed by the random sampling of values for the fast variables to be used to randomly sample a slow reaction. With the current paper, we try to address the central, averaging problem by giving exact equilibrium distributions and thereby exact expectations of the slow-scale propensities for as many systems as possible. In the electronic supplementary material, the embedding of an analytic equilibrium distribution into stochastic simulation is demonstrated using the above example.

## 4. Fast dynamics with quasi-steady-state assumption

We turn to the discussion of biochemical motifs where the equilibrium distribution can be computed analytically. Throughout the paper, all the reactions in the examples are fast. Other, slow reactions (possibly on multiple slower time scales) are omitted and are not subject of our interest. In each example, due to the ergodicity of the processes concerned, restricted to the communicating classes of the state spaces, the equilibrium distribution is unique. Where there are multiple communicating classes, the equilibrium distribution is a mixture of the equilibrium distributions on the individual communicating classes.

The success of these calculations hinges on a tractable state space, not on the form of the propensities. Neither the kinetics needs to be mass action, nor the reactions at most bimolecular. A crucial observation is that exact calculation can be possible even when the propensities are nonlinear. This is remarkable since, generally, even one nonlinear propensity function turns the transient time evolution of moments non-computable (the moment equations are not a finite set of ODEs in that case, see [37]). In our examples, the state spaces are simple, in most cases finite. In the finite cases certainly, the full chemical master equation (2.1) can be propagated, at least numerically. From that, even the time evolution of the moments is available. If the state space is infinite and some propensities are nonlinear, taking expectations first and then passing to infinity with time is possible through approximative moment closure methods [38,39].

### 4.1. Path-like state space

The first examples correspond to state spaces arranged along a line, with states naturally indexed by 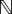 or {0, 1,…, *N*}. Transitions from a general state 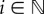 (*i* ≥ 1) are only allowed to neighbours *i* ± 1, and from *i* = 0 only to 1. In the finite case, from *i* = *N*, a transition is only allowed to *N* − 1.

#### 4.1.1. Infinite path

Consider the so-called *birth–death process*, where individuals are added to the system at rate *κ*_b_ and are removed with intensity proportional to the number of individuals present, with proportionality constant *κ*_d_. This is a model for the constitutive production and mutually independent degradation of a chemical species, e.g. an mRNA or a protein:
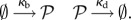


It is well known that the equilibrium distribution is the Poisson distribution with parameter *κ*_b_/*κ*_d_,
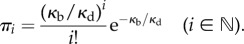


This is verified by writing out the right-hand side of the chemical master equation, first for *i* = 0
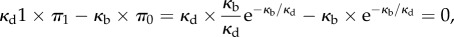
and for a general *i* ≥ 1,
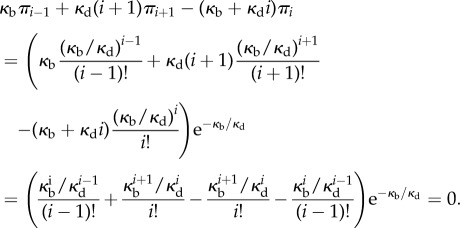


In fact, in this case of a linearly ordered state space, in equilibrium the stronger *detailed balance* condition must hold for any two neighbouring states
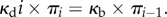


The Poisson distribution results as a consequence of this condition, and so does the equilibrium distribution of the finite-length path.

#### 4.1.2. Finite path

Let us have states indexed by {0, 1,…, *N*}, and transition rates
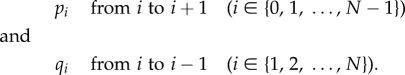


The detailed balance condition *q_i_*_+__1_*π_i_*_+1_ = *p_i_π*_*i*_, first with *i* = 0, yields *π*_1_ = *π*_0_*p*_0_/*q*_1_. The equilibrium distribution (*π*_0_, *π*_1_,…, *π*_*N*_)^T^ can be computed inductively in terms of *π*_0_ as (cf. [40])
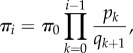
where *π*_0_ is determined by the normalization 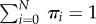. The evaluation of this *N* + 1-term sum may require numerical computation, which can be accelerated by a method suggested by Horner's scheme for polynomial evaluation
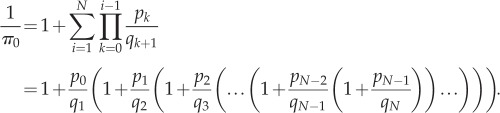


We demonstrate the applicability of this simple state space structure through three examples: isomerization, binding and dissociation of TFs to multiple copies of a gene and protein dimerization. This subclassification is also mathematically motivated: the order of reactions involved is different.

*Isomerization*. With two first-order reactions, one can model the isomerization of a molecule that has two stable states (e.g. conformational states) or the random opening and closing of an ion channel. Let the two states be denoted by 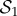 and 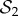,4.1
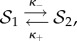
and the total molecule count *X*_1_ + *X*_2_ by *N*. Let *i* be a shorthand for *X*_1_, the number of 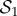 molecules, varying in {0, 1,…, *N*}. Then *p_i_* = *κ*_+_ (*N* − *i*) and *q_i_* = *κ*_−_*i*. The detailed balance condition for *i* ≤ *N* − 1 reads *κ*_−_ (*i* + 1)*π_i_*_+1_ = *κ*_+_ (*N* − *i*)*π_i_*. It is known that the solution is the binomial distribution with parameters *N* and *κ*_+_/(*κ*_−_ + *κ*_+_)
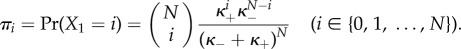


We verify this in the electronic supplementary material. Note that this is also a coarse model for the Goldbeter–Koshland switch [41], which is a system of covalent modifications facilitated by two converter enzymes, such as a phosphorylation–dephosphorylation system. If the two enzymes are present in fixed numbers 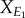 and 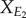, then this model applies with reaction rates 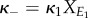 and 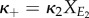 for some 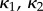.

*TF binding.* One second-order reaction of heterogeneous reactants and one first-order reaction suffice to model the regulation by TF binding of multiple copies of a gene (e.g. of a plasmid-borne bacterial gene). Let 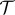 denote the TF, 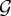 and 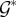 the free gene and the gene–TF complex, respectively. The total copy number of genes (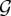 and 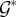 molecules) is *N*, and *T* is the total number of TFs (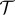 and 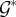 molecules). For simplicity, we require *N* ≤ *T*, but our derivation also gives the complementary case by interchanging the roles of the gene and the TF. The reaction system is
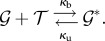
Let the state 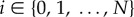 be the number of 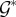, so that *p_i_* = *κ*_b_(*N* − *i*)(*T* − *i*) and *q_i_* = *κ*_u_*i*. The detailed balance condition for *i* ≤ *N* − 1 is now *κ*_u_(*i* + 1)*π*_*i*__+1_ = *κ*_b_(*N* − *i*)(*T* − *i*)*π*_*i*_. With induction, we can establish the equilibrium distribution up to normalization as4.2
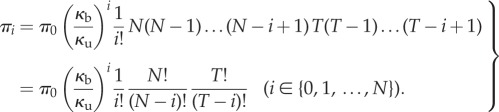
This scheme was used with a substrate and an enzyme in the place of, respectively, the TF and the gene to derive Michaelis and Menten's revered result in the stochastic setting [24]. We make the obvious remark that this is valid only if the catalysed reaction is slow and can be assumed to not be part of the fast time scale, as it does change the substrate and the enzyme counts.

*Dimerization.* Protein dimerization and dissociation is a biochemical system with one second-order reaction of homogeneous reactants and one first-order reaction. The state *i* will be the number of dimers 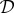, which we want to perform a random walk on state space {0, 1,*…*, *N*}. This requires that the total number of monomers, which is the number of free monomers *M* plus twice the number of dimers 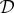, is 2*N* or 2*N* + 1. The reactions are given by4.3
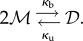


The transition rates are *p_i_* = *κ*_b_(2*N* − 2*i*)(2*N* − 2*i* − 1) in the even case (*p_i_* = *κ*_b_(2*N* + 1 − 2*i*)(2*N* − 2*i*) in the odd case) and *q_i_* = *κ*_u_*i*. The detailed balance equations are for *i* ≤ *N* − 1,
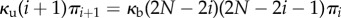
(*κ*_u_(*i* + 1)*π*_*i*__+__1_ = *κ*_b_(2*N* + 1 − 2*i*)(2*N* − 2*i*)*π*_*i*_, respectively). This gives the unnormalized equilibrium distribution for 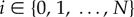 as
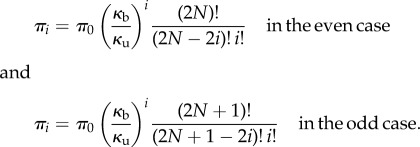
These replicate findings in [3, p. 5008] once their notations (*N*, *j*, *k_f_*, *k*_b_) are relabelled with (2*N*, *i*, 2*κ*_b_, *κ*_u_) in the even case (with (2*N* + 1, *i*, 2*κ*_b_, *κ*_u_) in the odd case); findings in [42, p. 1417] with (2*N*, *i*, *κ*_b_, *κ*_u_) in the even case ((2*N* + 1, *i*, *κ*_b_, *κ*_u_) in the odd case) in the places of (*n*, *i*, *c*_+_, *c*_−_); and findings in [18, p. 560] once we replace their (*z*_1_ + 2*z*_2_, *z*_2_, *κ*_9_, *κ*_10_) with (2*N*, *i*, *κ*_b_, *κ*_u_) in the even case (with (2*N* + 1, *i*, *κ*_b_, *κ*_u_) in the odd case).

#### 4.1.3. Two-state path

The general *finite path* case (§4.1.2) specializes to two states (*N* = 1) in a straightforward manner and gives the Bernoulli distribution as its equilibrium distribution. Indeed,
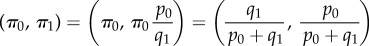
is obviously the unique solution.

For instance, the gene regulation model with only one gene present gives4.4
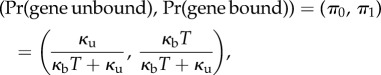
where one can recognize a Hill function with Hill coefficient 1.

#### 4.1.4. Three-state path

Another variation on the gene regulation models is a single gene that has two non-overlapping TF-binding sites (TFBS) such that a unique order in which the two TFBSs can be occupied by identical TFs is specified. Then the reaction system is
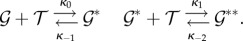


With general reaction rate constants, we allow for the modelling of cooperative binding. The transition rates are
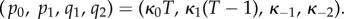


From the general result for finite-length paths, the equilibrium distribution is4.5
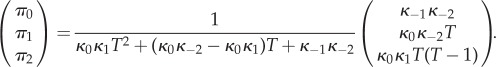


By evaluating *π*_0_*p*_0_/*q*_1_ and *π*_0_*p*_0_*p*_1_/(*q*_1_*q*_2_), one gets *π*_1_ and *π*_2_, as required.

Such a configuration might arise when the recruitment of the TF is facilitated by the preceding binding of one of *H* helper molecules. Then we have a reaction system
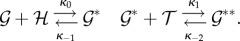


The transition rates are
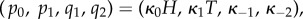
and the equilibrium distribution is
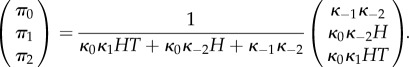


The equilibrium distribution for a four-state path model of a gene with three TFBSs was derived in [43]. The authors studied additional systems with the thermodynamic approach mentioned in our Introduction in connection with the study of Bintu *et al*. [27]. These systems are variants of a configuration where a gene has a number of non-identical TFBSs, one type is of interest, others are not. Two types of TFs bind to these, the first type to the TFBSs of interest with a so-called specific binding rate, and with a non-specific binding rate to the others. The second type of TFs binds all TFBSs with the non-specific binding rate. Maienschein-Cline *et al*. [44] used linear noise expansion to study equilibrium distributions in cases where *n* TFs, alternatively, *n*-mers bind to a gene (*n* ≥ 1).

### 4.2. Circular state space

Consider a state space where the chain of states {1, 2,…, *N*} closes into a circle and the transition rates are
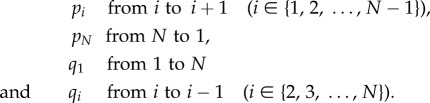


The equilibrium distribution of such Markov processes can be computed using
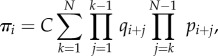
where *C* is a normalization constant, and for *i* > *N*, *p_i_* and *q_i_* should be interpreted as *p_i_*_−*N*_ and *q_i_*_−*N*_, respectively [45].

This formula for general Markov processes with a circular state space can be applied to important classes of biochemical systems. To make this point, we offer an example that is a variant of the three-state path, namely, a single gene with two non-overlapping TFBSs, where the identical TF molecules can bind to the TFBSs in any order. In terms of chemical reactions, this means
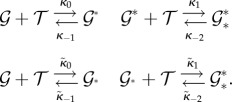


There are four states 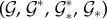, corresponding to the form in which the gene is found. If the total number of TFs is *T*, then the transition rates are given by 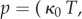 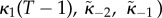 and 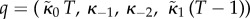. The theorem gives the following equilibrium distribution for a four-state cycle:4.6
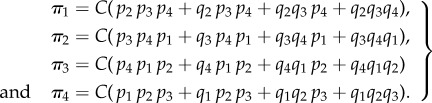


Before substitution, for simplicity, we assume 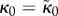, 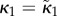, 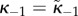 and 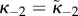. The model still enables cooperative binding as it is not ruled out that *κ*_0_ and *κ*_1_, or *κ*_−1_ and *κ*_−2_, are different. The previous formulae for the general case specialize to
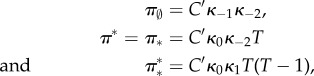
with *C’* = 2*C*(*κ*_−1_ + *κ*_1_(*T* − 1)). Thus, the solution is4.7
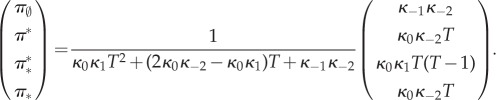
We verify these results in the electronic supplementary material.

The last two gene regulation models also suit enzyme–substrate complex formation with an enzyme that has two active sites. The same condition about time-scale separation and the non-occurrence of the catalysed reaction applies as for the Michaelis–Menten-type reaction (§4.1.2, TF binding).

*Hypercube, special case*. We can generalize the independent binding and unbinding of two TFs to a single gene to the case of the independent binding and unbinding of TFs to *N* binding sites of one gene. The state space is now an *N*-dimensional hypercube (instead of the square for *N* = 2) and can be indexed by 0 – 1 vectors of length *N*. Let there be *T* TFs in the system and we assume *N* ≤ *T*. Let 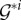 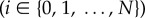 mean the entirety of the 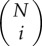 forms of the gene with *i* TFs bound. We derive the equilibrium distribution for these compounded states. The compounded states form a path-like state space with *N* + 1 states, no longer a hypercube. Between the compounded states transitions can be represented by reactions
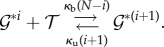


The forwards transition rate from 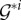 is *p_i_* = *κ*_b_(*N* − *i*)(*T* − *i*) (the binding rate for any one TF times the number of sites where it can bind times the number of free TFs). The backwards rate from 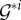 is *q_i_* = *κ*_u_*i* (the rate for unbinding times the number of occupied sites). One can recognize an already discussed reaction network hidden in this: the case of *N* genes independently binding one TF each. Thus, the equilibrium distribution is given by equation (4.2).

A generalization to occupancy-dependent reaction rate constants (to allow for cooperative binding) is also straightforward with *p_i_* = *κ_i_*(*N* − *i*)(*T* − *i*) and *q_i_* = *κ*_−*i*_*i*.

### 4.3. State space glued together from two graphs at one vertex

The next example is not a specific state space but a method to build more complex state spaces from well-understood ones. Assume that we have two continuous-time Markov processes on finite state spaces similar to those that have been presented so far. Let one process have states indexed by {1, 2,*…*, *r*} and known equilibrium distribution *π*^1^, while the other process has states {1, 2,…, *s*} and known equilibrium distribution *π*^2^.

One can get a new Markov process on the union of the two-state spaces by identifying state *r* of the first process and state 1 of the second process and keeping all transition rates unchanged that have been part of either chain. There is a transition defined between two states in the new process if and only if they originally belonged to the same state space and there was a transition between them in the respective original process. We claim that the new Markov process possesses the equilibrium distribution
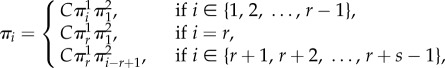
where states that belonged to the first process retained their original indices, states from the second process had their indices increased by *r* − 1 and 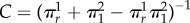. We verify this claim in the electronic supplementary material. Note that the second case could have been omitted as it is a special case of both the first and the third cases.

The combination of previous results paves the way to computing the equilibrium distribution in a reaction network whose state space consists of paths and cycles glued together. Gluing must always happen at one state at a time. For instance, cycles may only arise by adding the entire cycle in one step. It is possible to compute the equilibrium distribution when the two-state spaces are glued at two states simultaneously, but the procedure is complicated [46].

To demonstrate gluing in practice, we consider a gene with three non-overlapping TFBSs, one of which has the unique capacity to bind the first TF, the other two are not constrained in which order they bind the second and third TFs:
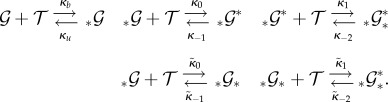


The combinatorial structure dictates that we glue this state space from a two-state state space 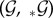 and a four-state circular state space 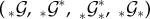. Here, as before, the total number of TFs is *T*, and we assume 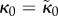, 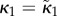, 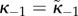 and 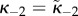. From previous results, it follows that the equilibrium distributions for the two separate processes are
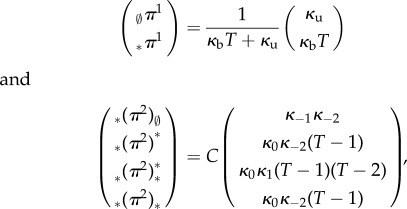
with 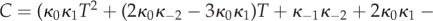 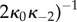. The equilibrium for the glued state space is
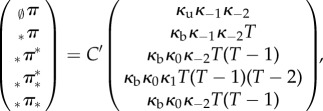
where
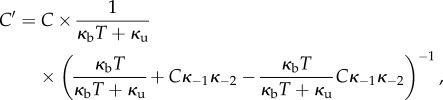
or *C’* can be expressed as the inverted sum of the five entries of the column vector.

A second example is best depicted by the mirror image of the previous state space. Here, two of the three non-overlapping TFBSs can bind the first two TFs in any order before the third TFBS binds the last TF:
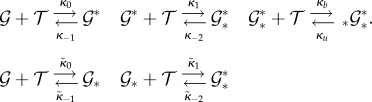


This network is discussed in the electronic supplementary material.

These two glued state spaces immediately lend themselves to applications where the binding of two TFs is preceded by the binding of a helper molecule, or where the binding of a single TF necessitates the previous recruitment of two helper molecules.

### 4.4. Kirchhoff's theorem or the Markov chain tree theorem

In addition to directly solving 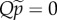, there is another general method to compute the equilibrium distribution of a continuous-time Markov process, which goes back to Kirchhoff [47]. A good exposition with background references is provided in [48], but the method had previously been developed elsewhere [49–51].

Consider the state space diagram as a directed graph 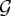. Whenever there is a transition (a directed edge) between two states, ensure that the transition in the opposite direction is also part of the graph, if necessary by including a zero intensity directed edge. For the moment, let us replace each pair of directed edges with a single undirected edge. We assume that this graph 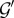 is connected. If this were not the case, then the argument would be applied to each component separately and the equilibrium distribution would arise as the mixture of the equilibriums on each component. Now consider all spanning trees of the connected, undirected graph 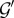.

For any state *i* of the state space and any spanning tree 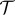 of 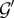, the *i*-directed spanning tree 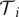 is defined by picking all the directed edges of the directed graph 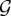 that correspond to the edges of 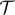 and in addition point towards *i*. This is a unique definition as a tree does not contain cycles. The claim is the following. The weight associated to state *i* in the equilibrium distribution is proportional to the sum over all *i*-directed spanning trees 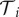 of products of all transition intensities on the directed edges of 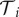. The proportionality constant is computed so that it normalizes the probability vector to one.

As a case study, it is easy to derive equation (4.6) for four-state cycles from Kirchhoff's result. Note that generally many of the intensities might be zero, hence many products will vanish in the sum. Whether this method is practically applicable in any particular system is dependent on the topological structure of the state space, and especially on whether it is easy to enlist all spanning trees.

### 4.5. Complex-balanced networks

A result, which has its roots in queuing theory and specifically is associated with Jackson networks, provides the equilibrium distribution of a large set of chemical reaction networks [52,53]. These networks are the complex-balanced ones, namely those whose deterministic, ODE model possesses a complex-balanced steady state.

It is necessary to introduce some of the terminology of chemical reaction network theory for this part. For more details throughout this section, see [34,54] in addition to [52].

A *complex* is any of the formal sums found on either side of a reaction. We refer to §2, where 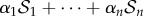 and 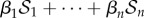 were presented as general complexes. These can be identified with the corresponding species vectors, (*α*_1_, … ,*α_n_*)^T^ and (*β*_1_, … ,*β*_*n*_)^T^, which we also call complexes.

For a specific reaction network, analogously to the stoichiometric matrix *ν* = [*ν*_1_ … *ν_m_*], we introduce the matrices 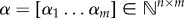 and 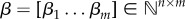 for the reactant and product sides, respectively. Here, *α_j_* = (*α*_1*j*_,…,*α_nj_*)^T^ and *β_j_* = (*β*_1*j*_,…,*β_nj_*)^T^ now refer to column vectors whose entries are the reactants and products of reaction 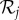, and *ν*_*j*_ = *β_j_* − *α_j_*.

Under the law of mass action, consider the reaction rate equation4.8
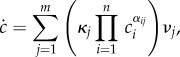
where *c* is the vector of species' concentrations. A *steady state* of this ODE is a point 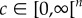, where 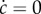. We call a deterministic, mass-action chemical reaction network *complex balanced*, if it satisfies a more stringent condition: it admits a strictly positive 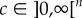 for which for every complex *ζ* of the reaction network
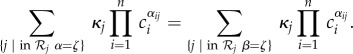


The sum on the left-hand side is over reactions for which *ζ* is a reactant complex, on the right-hand side over reactions for which *ζ* is a product complex. The equality expresses that each complex *ζ* is generated at the same rate as it is consumed.

We mentioned in §2 that conservation relations confine the state of the Markov process to stoichiometric compatibility classes and the equilibrium distribution can be determined on these classes separately. This also holds for the ODE model: equation (4.8) shows that the state can only evolve in directions that are contained in Im *ν*. Equivalently, when the stoichiometric matrix *ν* admits a left nullvector, this nullvector encodes a conserved quantity and the state space is partitioned into non-communicating compatibility classes according to the value of the conserved quantity. These compatibility classes are parallel affine hyperplanes that are orthogonal to the nullvector (or the intersections thereof, in the case of multiple linearly independent nullvectors).

It is the case that when a complex-balanced steady state exists, in fact there is exactly one in each stoichiometric compatibility class (and there are no other strictly positive steady states but these complex-balanced ones) [52, theorem 3.2]. We now state the result for complex-balanced networks.

**Theorem 4.1**

(Anderson *et al*. [52]). *Assume that the chemical reaction network has a complex-balanced steady state* 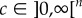
*. Then the Markov process model equipped with the intensities outlined in* §2 *has an equilibrium distribution that consists of the product of Poisson distributions: for each* 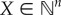,4.9
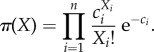

*If the state space* 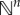 *is irreducible, then equation* (*4.9*) *is the unique equilibrium distribution. If it is not irreducible, then for each compatibility class* 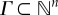, *the equilibrium distribution on* *Γ* *is, for each* 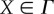,4.10
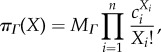
*with normalizing constant* *M_Γ_*.

Some guidance in interpretation will be beneficial. First, if there are different stoichiometric compatibility classes, to evaluate equation (4.10), the complex-balanced steady-state *c* can be freely chosen from any compatibility class. As long as for each *X* the same *c* is used, the so computed equilibrium distribution is independent of the choice of *c*. One can choose the steady state that makes the calculation the simplest, irrespective of the multiplicity of the molecules in the stochastic model. In other words, *c* is allowed to be from a compatibility class that is different to *Γ* in the stochastic model. *c* does not even need to be integer.

Second, in the irreducible case, the coordinates are independent random variables. Levine & Hwa [24] focused on this case and speculated about its biological role. However, in the non-irreducible case, independence of variables no longer holds.

The theorem is more general than is presented here: there is no need to restrict the reactant side to at most two interacting molecules in the stochastic mass-action case, and intensities more general than stochastic mass action are also admissible.

The *deficiency zero theorem*, Feinberg, Horn and Jackson's celebrated result of chemical reaction network theory, gives easily verifiable sufficient conditions for the existence of a complex-balanced steady state. Some further notions need to be introduced.

There is a unique directed graph associated to each chemical reaction network whose nodes are the complexes and in which there is a directed edge for each reaction channel from its reactant to its product complex. Each connected component of this graph is termed a *linkage class*. The network is called *weakly reversible*, if the linkage classes are strongly connected, which means that whenever there is a directed path from some *α* to an *α′*, there is also a directed path from *α′* to *α*. Finally, the *deficiency* of a reaction network is a non-negative integer, given by the number of its complexes (the number of nodes in the graph) minus the number of its linkage classes, minus the dimension of the stoichiometric subspace (dim Im *ν*).

Among other things, the deficiency zero theorem claims that if a chemical reaction network follows the dynamics (4.8), is weakly reversible, and has deficiency zero, then (regardless of the positive values of the rate constants *κ*) there exists a strictly positive, complex-balanced steady state in each stoichiometric compatibility class. The corollary of this theorem is that the product-form equilibrium distribution formula can be applied in networks that are deficiency zero and weakly reversible.

The isomerization model (4.1) was used in [52] as a demonstrative example for both the deficiency zero theorem and the result. The isomerization model also exposes that independence does not follow from a product-form equilibrium distribution. Other case studies of [52] include first-order reaction networks and certain enzymatic reactions. Zhang [55] used this result for a filament polymerization model.

The dimerization model (4.3) also satisfies the conditions of the deficiency zero theorem. The resulting product-form equilibrium distribution is equivalent to the originally presented distribution. The example in §4.6 with dimer TFs is another application of this corollary.

### 4.6. Ladder-shaped state space

**Figure 1.**
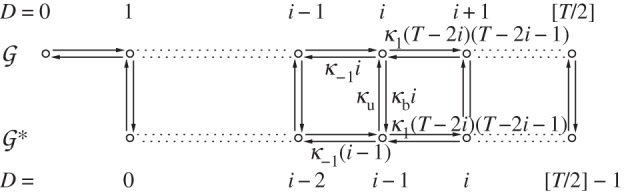
State space and transitions in the gene regulation mechanism with dimer TFs.

By a ladder-shaped state space, we mean a state space that can be indexed by 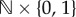 or {0, 1, 2,…, *N*} × {0, 1} 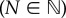: two parallel sides connected by edges at equally spaced intervals, perpendicularly to the parallel sides (cf. figure 1).

Peccoud & Ycart [56] considered a randomly activating and deactivating gene and the resulting mRNA or protein production and degradation4.11
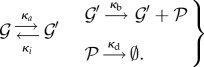
Hornos *et al.* [57] studied the expression of a self-repressing gene
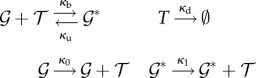
(*κ*_0_ > *κ*_1_). Grima *et al*. [58] corrected an oversight of that paper, which, incidentally, added diagonal transitions to the ladder. Visco *et al*. [59] and Vandecan & Blossey [60] studied three further similar systems.

Starting with [56], the standard technique to treat ladder-shaped state spaces has been to introduce two generating functions,
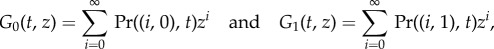
where *G*_0_ is for the states with inactive gene, and *G*_1_ is for the active gene. By multiplying by *z^i^* the chemical master equations for *∂*/*∂t*Pr((*i*, 0), *t*) and *∂/∂t*Pr((*i*, 1), *t*), and by summing for all *i*, a system of two partial differential equations (PDEs) for the two generating functions is derived. These can be solved, with a computer algebra system, to retrieve the time evolution of *G*_0_(*t*, *z*) + *G*_1_(*t*, *z*), and the mean protein copy number and variance. The two analogous generating functions of the equilibrium distribution give a system of two first-order ODEs, which can be solved in terms of the confluent hypergeometric function. The model (4.11) has been used to interpret experimental observations in Chinese hamster ovary cells [61]. The work by Iyer-Biswas *et al*. [62] extended the theoretical results and studied how variations in *κ_a_* and *κ_i_* influence transcript distribution.

Our focus will be on a gene regulation model with dimer TFs (figure 1)
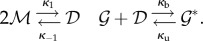
The approach we take avoids the established technique because in this finite state space, the generating functions are polynomials, and as there is no possibility to create a dimer when the dimer count is already maximal, there is a term missing from the right-hand sides of the PDEs needed to express everything directly in terms of the differentials. This term, which corresponds to the probability of maximal dimer count, can be reintroduced in terms of the derivative of the generating function of order maximal dimer count. However, the resulting PDE has a very high order. Instead of probability generating functions, we use the lesser-used product-form equilibrium distribution (theorem 4.1).

Let the total number of monomers *T* available to become TFs be constant on the fast time scale. It is assumed that there is just one copy of the gene: *G* + G* = 1. The variables (*M*, *D*, *G*, G*)^T^ satisfy the conservation relation *T* = *M* + 2*D* + 2G*. The state space is
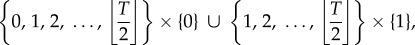
where the first coordinate is the total number of dimers (whether free or bound), the second coordinate is the state of the gene (free or bound), and transitions occur solely parallel to the axes. Note that one side of this ladder is longer than the other.

It is immediate that the network is reversible, therefore weakly reversible. It possesses four complexes, two linkage classes, and the reactions generate a two-dimensional stoichiometric subspace. Therefore, the deficiency zero theorem applies and the complex-balanced steady state 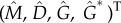 allows one to call on theorem 4.1.

Equation (4.10) is used due to the conservation relations. We compute the marginal probabilities of the gene being bound or free. The two corresponding sums over the different dimer counts have terms both falling and increasing in the summation index. These sums are treated as Cauchy products. They are estimated by Cauchy's coefficient formula applied to the resulting generating function, computed by the saddle-point method [63]. While the lengthy calculations are relegated to the electronic supplementary material (together with a verification by numerical simulation), we present the result here4.12
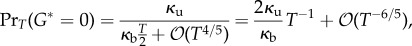
while Pr*_T_*(G* = 1) can be computed as 1 − Pr*_T_*(G* = 0).

This formula must be interpreted with due caution. First, note that the method is not sensitive enough to capture the dependence of Pr*_T_*(G* = 0) on *κ*_1_ and *κ*_−1_. This is heuristically explained by the fact that the steady-state number of dimers (in the ODE) is (see the electronic supplementary material)4.13
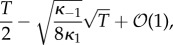
which is almost the maximum possible, *T*/2, corrected by a term of order 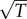 that is dependent on the constants *κ*_1_ and *κ*_−1_ but which is absorbed by the high error terms that are unavoidable with this method. Fundamentally, equation (4.12) is very similar to equation (4.4) with the number of TFs set to *T*/2. Second, note that the formula might well predict Pr*_T_*(G* = 0) > 1, depending on *κ*_b_ and *κ*_u_, but increasing *T* will always remedy this problem.

There is also a third method for similar calculations on ladder-shaped state spaces [42,64]. It is a distant relative of the method of §4.1.2 in that it is also iterative, but the computations are done on 2 × 2-matrices. One of the models of Fournier *et al*. [42, §4] is a gene regulatory system with fast dimerization of TFs, which was studied with a plethora of interesting techniques. However, no asymptotic formula like equation (4.12) was provided as the necessary iterations were only possible with numerical calculation.

## 5. A comparison of four gene regulatory systems

The preceding calculations enable the comparison of four simple gene regulation mechanisms. We present in each case the probability of the total occupation of the TFBSs of a single gene by TFs as a function of the number of TFs present. This response to the signal of the abundance of TFs is expressed by rational functions in the examples. For ease of comparison, here they will be converted to power series. With the exception of the dimer TFs, series expansions of arbitrary precision are achievable.

If a gene can bind one of *T* TFs, then from equation (4.4)5.1
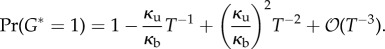


If the gene can bind one of several dimer TFs formed from *T* monomers, then equation (4.12) implies5.2
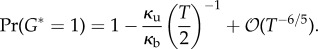


If the gene can bind two of *T* TFs sequentially, then by equation (4.5)5.3
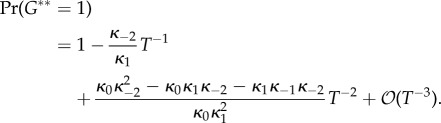


If the gene can bind two of *T* TFs independently, then by equation (4.7)5.4
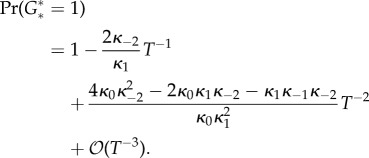


From the comments around the indicative count of dimers (4.13), it is not surprising that the dimer TF formula (5.2) mimics the single monomer TF formula (5.1) with *T*/2 TFs substituted in.

The similarity between formulae (5.3) and (5.4) is also clear. It is noteworthy that the leading terms are dependent on *κ*_1_ and *κ*_−2_ only. The factor 2 next to *κ*_−2_ in equation (5.4) has to do with the TFs being allowed to be released independently with rate *κ*_−2_. Similarly, in the independent case the free gene has twice the propensity to transition into the intermittent state with one TF bound compared with the sequential case. If both *κ*_0_ and *κ*_−2_ are divided by 2 in the independent case and the probabilities for the two intermittent states are summed, then the equilibrium distribution becomes identical to that of the sequential case.

## 6. Discussion

The most immediate application of the results of this paper is in stochastic simulation. The analytically computed exact equilibrium distributions can immensely speed up simulation (cf. §3). The tractability of these computations depends primarily on the graph structure of the state space, whereas the type of kinetics and the order of reactions play a lesser role.

In addition to the computational aspects, the theoretical implications are just as exciting. The analytical formulae can be used to study and compare regulatory elements more robustly and reliably than with simulation. We demonstrated this by studying the probability of the total occupation of the TFBS of a single gene by TFs in various set-ups. This is closely correlated to protein expression rates. The responses of different gene regulatory systems to TF counts, in the cases when quick gene expression response is required or when the TFs are metabolically expensive, might be a design consideration for synthetic biological applications, or a question of interest for basic research into engineering principles in gene regulation.

Our focus has been on the asymptotic behaviour of these probabilities as the number of TFs tends to infinity. In future work, we plan to study the behaviour at modest TF numbers, especially how sharp (how ‘switch-like’) the transitions are from the largely unbound to the largely bound form of the gene. We wish to know to what extent the tail determines the slope in the middle. This requires a finer exploration of the role that the rate constants play. We foresee drawing a parallel between these results for stochastic reaction models and the formalism and language of the traditionally deterministic, ODE-based Hill functions [65].

## Supplementary Material

Supporting information with additional details of calculations

## Supplementary Material

Mathematica 8 notebook with the detailed calculation for the gene regulatory system with dimer transcription factors

## Supplementary Material

Mathematica 8 notebook with series expansions of the full occupation probabilities of gene regulatory systems

## Data accessibility

An appendix and Mathematica 8 (Wolfram Research, Inc.) notebooks with additional details of calculations form part of this publication.
