# Supplementary material for: Equilibrium distributions of simple biochemical reaction systems for time-scale separation in stochastic reaction networks
Source: J R Soc Interface. 2014 Aug 6;11(97):20140054. doi: 10.1098/rsif.2014.0054 (PMC4208355; doi:10.1098/rsif.2014.0054)
Supplement: Supporting information with additional details of calculations [file rsif20140054supp1.pdf]

Equilibrium distributions of simple biochemical reaction systems for time-scale separation in stochastic reaction networks
Supporting information

Bence Mélykúti[*],[†] João P. Hespanha[‡], Mustafa Khammash[§]

6th June 2014

Connecting fast and slow time scales: A demonstration

To offer a small case study of integrating an analytic equilibrium distribution into stochastic simulation, we detail the gene regulatory system of *Connecting fast and slow time scales* of the main text. We let the increase in the abundance of transcription factors $\mathcal{T}$ trigger mRNA transcription from a target gene $\mathcal{G}$:

$$\emptyset \xrightarrow{\kappa_a} \mathcal{T} \qquad \mathcal{G} + \mathcal{T} \underset{\kappa_u}{\overset{\kappa_b}{\rightleftarrows}} \mathcal{G}^*$$

$$\mathcal{G}^* \xrightarrow{a_b} \mathcal{G}^* + \mathcal{M} \qquad \mathcal{M} \xrightarrow{\kappa_d} \emptyset.$$

If no TF is bound to the gene, zero transcription rate is assumed ($a_u = 0$). The variables are the gene count $X_{\mathcal{G}}$, the gene–TF complex count $X_{\mathcal{G}^*}$, the free TF count $X_{\mathcal{T}}$ and the mRNA count $X_{\mathcal{M}}$. Their initial states are given by

$$X(0) = (X_{\mathcal{G}}(0), X_{\mathcal{G}^*}(0), X_{\mathcal{T}}(0), X_{\mathcal{M}}(0))^{\mathrm{T}} = (1, 0, 0, 0)^{\mathrm{T}}.$$

[*]Department of Mathematical Stochastics, University of Freiburg, Eckerstr. 1, 79104 Freiburg, Germany

[†]Centre for Biological Systems Analysis (ZBSA), University of Freiburg, Habsburgerstr. 49, 79104 Freiburg, Germany

[‡]Electrical & Computer Engineering, Harold Frank Hall, University of California, Santa Barbara, CA 93106-9560, USA

[§]Department of Biosystems Science and Engineering, ETH Zürich, Mattenstrasse 26, 4058 Basel, Switzerland

The rate constants

$$\kappa_a = 0.02 \text{ s}^{-1},$$
$$\kappa_b = 0.2 \text{ s}^{-1},$$
$$\kappa_u = 5 \text{ s}^{-1},$$
$$a_b = 0.01 \text{ s}^{-1},$$
$$\kappa_d = 0.0005 \text{ s}^{-1}$$

are chosen for this example such that the dynamics has two well-separated time scales: the association and dissociation of the TF and the gene are fast reactions, while the arrival of TFs, and the creation and degradation of mRNA are slow reactions. We see that the total number of TFs

$$T(t) = X_{\mathcal{G}^*}(t) + X_{\mathcal{T}}(t)$$

is increasing in time, but only on the slow time scale. On the fast time scale, under the quasi-steady-state assumption, it is stipulated to be constant. The total number of genes

$$\widehat{G}(t) = X_{\mathcal{G}}(t) + X_{\mathcal{G}^*}(t) = 1$$

is constant in the absolute sense.

The model under the QSSA is described by variables $\widetilde{X}(t) = (\widehat{G}(t), T(t), X'_{\mathcal{M}}(t))^{\mathrm{T}}$ and consists of reactions

$$\emptyset \xrightarrow{\kappa_a} \mathcal{T}$$

$$\widehat{\mathcal{G}} \xrightarrow{a(\widetilde{X})} \widehat{\mathcal{G}} + \mathcal{M} \qquad \qquad \mathcal{M} \xrightarrow{\kappa_d} \emptyset.$$

In this approximative model, $\widehat{G}(t) = 1$ for all $t \geq 0$, and

$$\widetilde{X}(0) = (\widehat{G}(0), T(0), X'_{\mathcal{M}}(0))^{\mathrm{T}} = (1, 0, 0)^{\mathrm{T}}.$$

Since this is an approximation, the mRNA counts will differ slightly between the two models. The hope is that the distributions of $X_{\mathcal{M}}(t)$ and $X'_{\mathcal{M}}(t)$ are very similar for all $t \geq 0$. (Even the distributions of the trajectories should be close.) The effective transcription rate is

$$a(\widetilde{X}(t)) = a_u \mathrm{P}_{\mathrm{QSS}}(\mathcal{G} \mid \widetilde{X}(t)) + a_b \mathrm{P}_{\mathrm{QSS}}(\mathcal{G}^* \mid \widetilde{X}(t)) = a_b \frac{\kappa_b T(t)}{\kappa_b T(t) + \kappa_u},$$

due to $a_u = 0$ and the section *Two-state path* of the main text.

The numerical simulations by Gillespie's *stochastic simulation algorithm* [1] were implemented in GNU Octave with code that is compatible with Matlab (The MathWorks, Inc.). For both the complete and the QSSA models, 3000 sample trajectories

were generated over a time interval of length 7200 s = 2 h. These trajectories were sampled every 10 s and this data was stored on disk.

Figure 1 shows the mean and mean $\pm$ standard deviation curves over time for the total TF count in the complete and in the QSSA model, together with the mean and mean $\pm$ standard deviation curves for the mRNA count in the two models. The total TF counts in the two models should be nearly identical since the arrival process is upstream from the QSS approximation. Any difference is due to sampling error (also known as Monte Carlo error). It is the mRNA count where the models are expected to differ slightly, and the size of this difference is the measure of the inaccuracy of the approximation.

The arrival of TFs is a Poisson process, therefore we expect their total number to increase about linearly, and at 2 h, to be Poisson distributed with parameter $\kappa_a \times 7200\ \text{s} = 144$. The data corroborates this prediction: in the complete model, we observed 144.09 ± 11.85 TF molecules (free and bound), and in the QSSA model, 144.22 ± 11.91.

The mRNA count at the end of the simulated time interval was 15.30 ± 3.91 in the complete model, and 15.46 ± 4.02 in the QSSA model. The QSS approximation is very accurate for this model with these particular parameter values.

In the complete model, at the end of the 2 h time interval, the gene was in the complex form in 84.7% of the 3000 cases. If the gene were always in the on state, then the mRNA population would reach balance at $a_b/\kappa_d = 20$. (This would be the mean of its Poisson distribution, cf. section *Infinite path.*) Thus the mean mRNA count was expected to be observed slightly below this value.

Generating the 3000 sample trajectories for the complete model took 8.1 h on a personal computer (with a 2.8 GHz Intel Core i7-2640M dual-core processor and 4 GB RAM), while the same task was finished in less than 2 min for the QSSA model. The subsampling at 10 s intervals was introduced to avoid unnecessarily discriminating against the complete model by keeping in memory and then saving the larger amount of data it generated. In the complete model, on average, one trajectory consisted of 48139.2 reactions (of which, on average, 23957.95, respectively, 23957.11 reactions were the formation and dissociation of the gene-TF complex), and in the QSSA model, an average trajectory consisted of 224.86 reactions. In the complete model, on average, 47.69 reactions created an mRNA, 32.39 reactions degraded one. In the QSSA model, the corresponding numbers were 48.05 and 32.59 reactions, respectively. Notice that the difference $23957.95 - 23957.11 = 0.847$ between complex formation and dissociation is just the proportion of trajectories in which the gene was found in complex form at the end.

In general, the running time saved and the accuracy of the QSSA are closely linked to how well the fast and the slow time scales are separated, which in turn is related to the magnitudes of the rate constants and molecular counts.

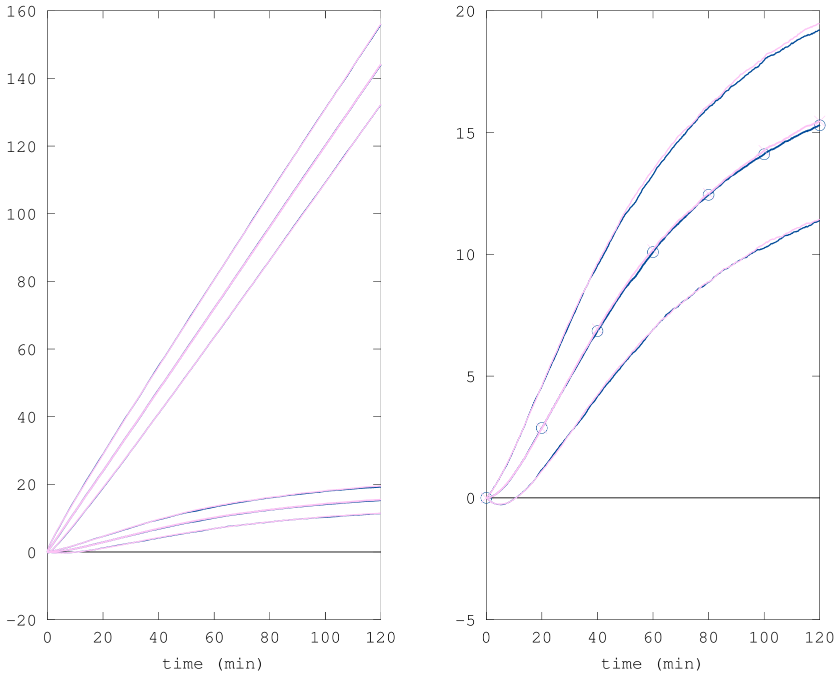

Figure 1: (*left panel*) Simulation results of the total TF count (larger values) and the mRNA count (smaller values) in the complete model (in blue) and in the QSSA model (in pink). The displayed triplets of curves are the mean and the mean ± standard deviation. (*right panel*) mRNA counts only, in the complete model (blue lines and circles for the mean values at the displayed times) and in the QSSA model (pink lines and crosses for the mean values at the displayed times).

Detailed balance for isomerisation

We verify that the binomial distribution satisfies the detailed balance equations $\kappa_-(i+1)\pi_{i+1} = \kappa_+(N-i)\pi_i$ ($i \in \{0, 1, \ldots, N-1\}$) for the isomerisation model:

$$\begin{aligned}
\kappa_-(i+1)\pi_{i+1} &= \kappa_-(i+1)\,\frac{N!}{(i+1)!(N-(i+1))!}\,\frac{\kappa_+^{i+1}\kappa_-^{N-(i+1)}}{(\kappa_- + \kappa_+)^N} \\
&= \frac{N!}{i!(N-i-1)!}\,\frac{\kappa_+^{i+1}\kappa_-^{N-i}}{(\kappa_- + \kappa_+)^N} \\
&= \kappa_+(N-i)\,\frac{N!}{i!(N-i)!}\,\frac{\kappa_+^{i}\kappa_-^{N-i}}{(\kappa_- + \kappa_+)^N} \\
&= \kappa_+(N-i)\pi_i.
\end{aligned}$$

The first and the last equalities hold by definition, the second and the third steps are algebraic rearrangements.

Verification of the equilibrium conditions in the circular state space model of cooperative binding

We verify both the general formula for four-state circular state spaces and the application to the independent binding of two TFs to a gene without relying on Adan and Resing's result [2].

First, we prove for the general, circular Markov process that a general state i is in equilibrium if all states are drawn from the proposed distribution π. This is equivalent to $p_{i-1}\pi_{i-1} + q_{i+1}\pi_{i+1} = p_i\pi_i + q_i\pi_i$ for any $i \in \{1, 2, 3, 4\}$. We simply substitute the proposed solution into each of these four terms to get

$$\begin{aligned}
p_{i-1}\pi_{i-1} &= p_{i+3}C(p_ip_{i+1}p_{i+2} + q_ip_{i+1}p_{i+2} + q_iq_{i+1}p_{i+2} + q_iq_{i+1}q_{i+2}), \\
q_{i+1}\pi_{i+1} &= q_{i+1}C(p_{i+2}p_{i+3}p_i + q_{i+2}p_{i+3}p_i + q_{i+2}q_{i+3}p_i + q_{i+2}q_{i+3}q_i), \\
p_i\pi_i &= p_iC(p_{i+1}p_{i+2}p_{i+3} + q_{i+1}p_{i+2}p_{i+3} + q_{i+1}q_{i+2}p_{i+3} + q_{i+1}q_{i+2}q_{i+3}), \\
q_i\pi_i &= q_iC(p_{i+1}p_{i+2}p_{i+3} + q_{i+1}p_{i+2}p_{i+3} + q_{i+1}q_{i+2}p_{i+3} + q_{i+1}q_{i+2}q_{i+3}).
\end{aligned}$$

Here, as before, indices are to be interpreted modulo 4. Accordingly, we have already written out indices $i-1$ as $i+3$ (in the first equality) and $i+4$ as i (in the second equality). If one expands out the products, there is a one-to-one correspondence between the eight terms in $p_{i-1}\pi_{i-1} + q_{i+1}\pi_{i+1}$ and eight equal counterparts in $p_i\pi_i + q_i\pi_i$.

Second, the equilibrium distribution for the gene regulation model will be validated

if $Q\pi = 0$ is proven for

$$Q = \begin{bmatrix} -2\kappa_0 T & \kappa_{-1} & 0 & \kappa_{-1} \\ \kappa_0 T & -\kappa_1(T-1) - \kappa_{-1} & \kappa_{-2} & 0 \\ 0 & \kappa_1(T-1) & -2\kappa_{-2} & \kappa_1(T-1) \\ \kappa_0 T & 0 & \kappa_{-2} & -\kappa_{-1} - \kappa_1(T-1) \end{bmatrix},$$

$$\pi = (\pi_\emptyset, \pi^*, \pi^*_*, \pi_*)^{\mathrm{T}}.$$

In fact, it is enough to compute $Q\,(C')^{-1}\pi$ ($Q\pi$ up to a constant scalar factor), which gives the following:

$$\begin{aligned}
(Q\,(C')^{-1}\pi)_1 &= -2\kappa_0 T\kappa_{-1}\kappa_{-2} + \kappa_{-1}\kappa_0\kappa_{-2}T + 0 + \kappa_{-1}\kappa_0\kappa_{-2}T = 0,\\
(Q\,(C')^{-1}\pi)_2 &= \kappa_0 T\kappa_{-1}\kappa_{-2} - (\kappa_1(T-1) + \kappa_{-1})\kappa_0\kappa_{-2}T + \kappa_{-2}\kappa_0\kappa_1 T(T-1) + 0 = 0,\\
(Q\,(C')^{-1}\pi)_3 &= 0 + \kappa_1(T-1)\kappa_0\kappa_{-2}T - 2\kappa_{-2}\kappa_0\kappa_1 T(T-1) + \kappa_1(T-1)\kappa_0\kappa_{-2}T = 0,\\
(Q\,(C')^{-1}\pi)_4 &= \kappa_0 T\kappa_{-1}\kappa_{-2} + 0 + \kappa_{-2}\kappa_0\kappa_1 T(T-1) - (\kappa_{-1} + \kappa_1(T-1))\kappa_0\kappa_{-2}T = 0.
\end{aligned}$$

This completes the verification of the two equilibrium distributions.

Verification of the equilibrium conditions for the Markov process with the glued state space

The correctness of the proposed distribution is proved in three parts. First, that it is nonnegative. All π^1_i and π^2_i are nonnegative and at most one, since these are distributions. Therefore $\pi^1_r \geq \pi^1_r\pi^2_1$, and $\pi^1_r + \pi^2_1 - \pi^1_r\pi^2_1 \geq 0$.

The following calculation proves that π sums to one:

$$\begin{aligned}
\frac{1}{C}\sum_{i=1}^{r+s-1}\pi_i &= \sum_{i=1}^{r}\pi^1_i\pi^2_1 + \sum_{i=r+1}^{r+s-1}\pi^1_r\pi^2_{i-r+1}\\
&= 1\pi^2_1 + \sum_{i=2}^{s}\pi^1_r\pi^2_i\\
&= \pi^2_1 + \pi^1_r(1-\pi^2_1)\\
&= \pi^2_1 + \pi^1_r - \pi^1_r\pi^2_1.
\end{aligned}$$

The first equality is nothing but the definition. The second equality is a consequence of π^1 being a distribution and of a return to the original indices in the second part of the state space. The third equality reflects that π^2 is a distribution. Multiplying the first and the last lines by C proves $\sum_{i=1}^{r+s-1}\pi_i = 1$.

The third part is to prove $Q\pi = 0$. Here Q is almost a block-diagonal matrix formed from Q^1 and Q^2. The difference is that there is an overlap at the glued state where the

diagonal elements (negative exit rates) are added:

$$Q_{ij} = \begin{cases} Q^1_{ij}, & \text{for } i \in \{1,2,\ldots,r-1\},\ j \in \{1,2,\ldots,r\}, \\ 0, & \text{for } i \in \{1,2,\ldots,r-1\},\ j \in \{r+1,r+2,\ldots,r+s-1\}, \\ Q^1_{rj}, & \text{for } i = r,\ j \in \{1,2,\ldots,r-1\}, \\ Q^1_{rr} + Q^2_{11}, & \text{for } i = r,\ j = r, \\ Q^2_{1,j-r+1}, & \text{for } i = r,\ j \in \{r+1,r+2,\ldots,r+s-1\}, \\ 0, & \text{for } i \in \{r+1,r+2,\ldots,r+s-1\},\ j \in \{1,2,\ldots,r-1\}, \\ Q^2_{i-r+1,j-r+1}, & \text{for } i \in \{r+1,r+2,\ldots,r+s-1\},\ j \in \{r,r+1,\ldots,r+s-1\}. \end{cases}$$

We check $Q\pi = 0$ for three different cases with respect to row index. For $i \in \{1,2,\ldots,r-1\}$, by substituting the definitions of Q and π,

$$(QC^{-1}\pi)_i = (Q^1\pi^1)_i\,\pi^2_1 = 0\,\pi^2_1 = 0,$$

since π^1 is an equilibrium distribution for Q^1. Similarly, for $i \in \{r+1, r+2, \ldots, r+s-1\}$,

$$(QC^{-1}\pi)_i = \pi^1_r\,(Q^2\pi^2)_{i-r+1} = \pi^1_r\,0 = 0.$$

For $i = r$,

$$\begin{aligned}(QC^{-1}\pi)_r &= \sum_{k=1}^{r-1} Q^1_{rk}\pi^1_k\,\pi^2_1 + \left(Q^1_{rr} + Q^2_{11}\right)\pi^1_r\pi^2_1 + \sum_{k=r+1}^{r+s-1} \pi^1_r\,Q^2_{1,k-r+1}\pi^2_{k-r+1} \\ &= \pi^2_1 \sum_{k=1}^{r} Q^1_{rk}\pi^1_k + \pi^1_r \sum_{k=r}^{r+s-1} Q^2_{1,k-r+1}\pi^2_{k-r+1} \\ &= \pi^2_1\,0 + \pi^1_r\,0 = 0,\end{aligned}$$

and the proof is complete.

A second application of glued state spaces

The state space of this example is the mirror image of the first example of glued state spaces in the main text. Here two of the three non-overlapping TFBSs can bind the first two TFs independently before the third TFBS binds the last TF:

$$\mathcal{G} + \mathcal{T} \underset{\kappa_{-1}}{\overset{\kappa_0}{\rightleftarrows}} \mathcal{G}^* \qquad \mathcal{G}^* + \mathcal{T} \underset{\kappa_{-2}}{\overset{\kappa_1}{\rightleftarrows}} \mathcal{G}^*_* \qquad \mathcal{G}^*_* + \mathcal{T} \underset{\kappa_u}{\overset{\kappa_b}{\rightleftarrows}} {}_*\mathcal{G}^*_*.$$

$$\mathcal{G} + \mathcal{T} \underset{\widetilde{\kappa}_{-1}}{\overset{\widetilde{\kappa}_0}{\rightleftarrows}} \mathcal{G}_* \qquad \mathcal{G}_* + \mathcal{T} \underset{\widetilde{\kappa}_{-2}}{\overset{\widetilde{\kappa}_1}{\rightleftarrows}} \mathcal{G}^*_*$$

The state space is constructed by gluing the last state of $(\mathcal{G}_*, \mathcal{G}, \mathcal{G}^*, \mathcal{G}^*_*)$ to the first state of $(\mathcal{G}^*_*, {}_*\mathcal{G}^*_*)$. The assumptions $\kappa_0 = \widetilde{\kappa}_0$, $\kappa_1 = \widetilde{\kappa}_1$, $\kappa_{-1} = \widetilde{\kappa}_{-1}$, and $\kappa_{-2} = \widetilde{\kappa}_{-2}$ are still stipulated.

The equilibrium distribution on the first state space, $\left((\pi^1)_\emptyset, (\pi^1)^*, (\pi^1)^*_*, (\pi^1)_*\right)^{\mathrm{T}}$, is given by Equation (8) of the main text, whereas on the second state space by

$$\begin{pmatrix} {}_\emptyset(\pi^2)^*_* \\ {}_*(\pi^2)^*_* \end{pmatrix} = \frac{1}{\kappa_b(T-2)+\kappa_u} \begin{pmatrix} \kappa_u \\ \kappa_b(T-2) \end{pmatrix}.$$

Therefore the equilibrium distribution on the glued state space is

$$\begin{pmatrix} \pi_* \\ \pi_\emptyset \\ \pi^* \\ \pi^*_* \\ {}_*\pi^*_* \end{pmatrix} = C \begin{pmatrix} \kappa_0\kappa_{-2}\kappa_u T \\ \kappa_{-1}\kappa_{-2}\kappa_u \\ \kappa_0\kappa_{-2}\kappa_u T \\ \kappa_0\kappa_1\kappa_u T(T-1) \\ \kappa_0\kappa_1\kappa_b T(T-1)(T-2) \end{pmatrix},$$

where C can be given as the inverted sum of the five entries of the right-hand-side vector.

Ladder-shaped state space, dimer transcription factors

We will need a number of well-known identities and estimates.

Proposition 1 1. For any $\varepsilon \in]-1,1[$,

$$\sqrt{1+\varepsilon} = 1 + \frac{\varepsilon}{2} + \mathcal{O}(\varepsilon^2). \tag{1}$$

2. (Cauchy's coefficient formula) Let $U \subseteq \mathbb{C}$ be an open set containing 0, $f : U \to \mathbb{C}$ an analytic (with different terminology, holomorphic) function and let γ be a simple loop around 0 (that is, a null-homotopic, one-to-one, closed curve) that is positively oriented. Then the coefficient $[z^n]f(z) := f_n$ in the power series expansion $f(z) = \sum_{n=0}^{\infty} f_n z^n$ admits the representation

$$[z^n]f(z) = \frac{1}{2\pi i}\int_\gamma \frac{f(z)}{z^{n+1}}\,\mathrm{d}z. \tag{2}$$

3. For any $\vartheta \in \mathbb{R}$,

$$\cos\vartheta = 1 - \frac{\vartheta^2}{2!} + \mathcal{O}(\vartheta^4). \tag{3}$$

4.

$$\int_{-\infty}^{\infty} e^{-t^2/2}\,\mathrm{d}t = \sqrt{2\pi}. \tag{4}$$

5. For any $c \geq 0$,

$$\int_c^{\infty} e^{-t^2/2}\,\mathrm{d}t = \mathcal{O}(e^{-c^2/2}). \tag{5}$$

Proof The series expansions of the square root (1) and the cosine function (3) are standard [3, p. 15 and p. 74].

Cauchy's coefficient formula (2) is a central result of complex analysis and can be found, for instance, in [4, Theorem IV.4, p. 237].

Eq. (4) can be verified via the well-known form of the probability density function of the normal distribution.

The estimate (5) will be proven for $c \in [0, 1]$ and $c \geq 1$ separately.

$$\int_c^\infty e^{-t^2/2}\,\mathrm{d}t \leq \int_0^\infty e^{-t^2/2}\,\mathrm{d}t = \sqrt{\frac{\pi}{2}}$$

from Eq. (4). Further, for any $c \in [0, 1]$,

$$\sqrt{\frac{\pi}{2}} = \left(\sqrt{\frac{\pi}{2}} e^{1/2}\right) e^{-1/2} \leq \left(\sqrt{\frac{\pi}{2}} e^{1/2}\right) e^{-c^2/2}.$$

The case $c \geq 1$ is left. From

$$\left(e^{-t^2/2}\right)' = -te^{-t^2/2},$$

it follows

$$\int_c^\infty e^{-t^2/2}\,\mathrm{d}t = \frac{1}{c}\,c\int_c^\infty e^{-t^2/2}\,\mathrm{d}t \leq \frac{1}{c}\int_c^\infty te^{-t^2/2}\,\mathrm{d}t = \frac{1}{c}e^{-c^2/2} \leq e^{-c^2/2}. \qquad \square$$

Let us fix the total number of monomers to be $T \in \mathbb{N}$. $G + G^* = 1$ is also fixed. For this particular stoichiometric compatibility class, the product-form equilibrium distribution in terms of a complex-balanced steady state $(\hat{M}, \hat{D}, \hat{G}, \hat{G}^*)^{\mathrm{T}}$ is the following:

$$\pi_T\left((M,\, D,\, G,\, G^*)^{\mathrm{T}}\right) = \chi_{\{M=T-2D-2G^*,\, G=1-G^*\}} C_T \,\frac{\hat{M}^M}{M!}\,\frac{\hat{D}^D}{D!}\,\frac{\hat{G}^G}{G!}\,\frac{(\hat{G}^*)^{G^*}}{G^*!},$$

with T-dependent normalizing constant C_T [5].

Of interest to us are the probabilities that the gene is in the bound or in the unbound state. For motivation and brevity, we already include the result of a later calculation, Eq. (7), in the fourth equality:

$$\begin{aligned}
\mathrm{P}_T(G^* = 0) &= \sum_{\{(M,D,G,G^*)\,|\,G^*=0\}} \pi_T\left((M,\, D,\, G,\, G^*)^{\mathrm{T}}\right) \\
&= \sum_{\{(M,D,G,G^*)\,|\,G^*=0\}} \chi_{\{M=T-2D-2G^*,\, G=1-G^*\}} C_T \,\frac{\hat{M}^M}{M!}\,\frac{\hat{D}^D}{D!}\,\frac{\hat{G}^G}{G!}\,\frac{(\hat{G}^*)^{G^*}}{G^*!} \\
&= \sum_{D=0}^{\lfloor T/2 \rfloor} C_T \,\frac{(\hat{T} - 2\hat{D} - 2\hat{G}^*)^{T-2D}}{(T-2D)!}\,\frac{\hat{D}^D}{D!}\,\frac{\hat{G}^1}{1!}\,\frac{(\hat{G}^*)^0}{0!} \\
&= C_T \frac{\kappa_u}{\kappa_b \hat{D} + \kappa_u}\,\hat{G}_t \sum_{D=0}^{\lfloor T/2 \rfloor} \frac{(\hat{T} - 2\hat{D} - 2\hat{G}^*)^{T-2D}}{(T-2D)!}\,\frac{\hat{D}^D}{D!}.
\end{aligned}$$

Similarly,

$$
\begin{aligned}
\mathrm{P}_T(G^*=1) &= \sum_{\{(M,D,G,G^*)\,|\,G^*=1\}} \pi_T\left((M,\,D,\,G,\,G^*)^{\mathrm{T}}\right) \\
&= \sum_{\{(M,D,G,G^*)\,|\,G^*=1\}} \chi_{\{M=T-2D-2G^*,\,G=1-G^*\}} C_T \frac{\hat{M}^M}{M!}\frac{\hat{D}^D}{D!}\frac{\hat{G}^G}{G!}\frac{(\hat{G}^*)^{G^*}}{G^*!} \\
&= \sum_{D=0}^{\lfloor T/2\rfloor-1} C_T \frac{(\hat{T}-2\hat{D}-2\hat{G}^*)^{T-2D-2}}{(T-2D-2)!}\frac{\hat{D}^D}{D!}\frac{\hat{G}^0}{0!}\frac{(\hat{G}^*)^1}{1!} \\
&= C_T \frac{\kappa_b\hat{D}}{\kappa_b\hat{D}+\kappa_u}\hat{G}_t \sum_{D=0}^{\lfloor T/2\rfloor-1} \frac{(\hat{T}-2\hat{D}-2\hat{G}^*)^{T-2D-2}}{(T-2D-2)!}\frac{\hat{D}^D}{D!}.
\end{aligned}
$$

The summands consist of one factor having terms increasing in the summation index D and the other factor with terms decreasing in D. The sums will be represented as coefficients in the product of two power series. Consider the following calculation.

$$
\begin{aligned}
\exp\left(\hat{D}z^2+(\hat{T}-2\hat{D}-2\hat{G}^*)z\right) &= \sum_{k=0}^{\infty}\frac{\hat{D}^k z^{2k}}{k!}\sum_{\ell=0}^{\infty}\frac{(\hat{T}-2\hat{D}-2\hat{G}^*)^{\ell}z^{\ell}}{\ell!} \\
&= \sum_{n=0}^{\infty}\sum_{\{(k,\ell)\in\mathbb{N}^2\ |\ 2k+\ell=n\}}\frac{\hat{D}^k}{k!}\frac{(\hat{T}-2\hat{D}-2\hat{G}^*)^{\ell}}{\ell!}z^n \\
&= \sum_{n=0}^{\infty}\sum_{k=0}^{\lfloor n/2\rfloor}\frac{\hat{D}^k}{k!}\frac{(\hat{T}-2\hat{D}-2\hat{G}^*)^{n-2k}}{(n-2k)!}z^n,
\end{aligned}
$$

where the first equality follows from the series expansion of both factors of the factorised exponential function, in the second line this is written in terms of a Cauchy product of the two power series (the rearrangement is justified by the absolute convergence of both series), and the third equality uses $\ell = n-2k$. This argument immediately yields

$$
\begin{aligned}
\mathrm{P}_T(G^*=0) &= C_T\frac{\kappa_u}{\kappa_b\hat{D}+\kappa_u}\hat{G}_t\times[z^T]\exp\left(\hat{D}z^2+(\hat{T}-2\hat{D}-2\hat{G}^*)z\right), \\
\mathrm{P}_T(G^*=1) &= C_T\frac{\kappa_b\hat{D}}{\kappa_b\hat{D}+\kappa_u}\hat{G}_t\times[z^{T-2}]\exp\left(\hat{D}z^2+(\hat{T}-2\hat{D}-2\hat{G}^*)z\right).
\end{aligned}
$$

The unknown normalizing constant C_T can be removed by algebraic rearrangements,

$$
\begin{aligned}
\mathrm{P}_T(G^*=0) &= \frac{\mathrm{P}_T(G^*=0)}{\mathrm{P}_T(G^*=0)+\mathrm{P}_T(G^*=1)} = \frac{1}{1+\frac{\mathrm{P}_T(G^*=1)}{\mathrm{P}_T(G^*=0)}} \\
&= \frac{1}{1+\frac{\kappa_b\hat{D}\,[z^{T-2}]\exp\left(\hat{D}z^2+(\hat{T}-2\hat{D}-2\hat{G}^*)z\right)}{\kappa_u\,[z^T]\exp\left(\hat{D}z^2+(\hat{T}-2\hat{D}-2\hat{G}^*)z\right)}} \\
&= \frac{\kappa_u}{\kappa_u+\kappa_b\hat{D}\frac{[z^{T-2}]\exp\left(\hat{D}z^2+(\hat{T}-2\hat{D}-2\hat{G}^*)z\right)}{[z^T]\exp\left(\hat{D}z^2+(\hat{T}-2\hat{D}-2\hat{G}^*)z\right)}}. \qquad (6)
\end{aligned}
$$

For the sake of clearer notation, we introduce some new symbols. With

$$\sigma := \hat{M} = \hat{T} - 2\hat{D} - 2\hat{G}^*,$$
$$\tau := \hat{D},$$

the exponential function of interest becomes

$$F(z) = \exp\left(\tau z^2 + \sigma z\right),$$

and its power series expansion

$$F(z) = \sum_{n=0}^{\infty} f_n z^n.$$

Recall that the complex-balanced steady state in the product-form equilibrium distribution theorem (essentially, its stoichiometric compatibility class) can be chosen independently from the stoichiometric compatibility class in which we compute the equilibrium distribution of the stochastic model. When we want to emphasise the dependence of F on this choice, which is the choice of $\hat{T}$ and $\hat{G}_t := \hat{G} + \hat{G}^*$, we may write

$$F_{(\hat{T},\hat{G}_t)}(z) = \exp\left(\tau_{(\hat{T},\hat{G}_t)} z^2 + \sigma_{(\hat{T},\hat{G}_t)} z\right) = \sum_{n=0}^{\infty} f_{(\hat{T},\hat{G}_t),n} z^n.$$

Our aim becomes to compute $f_{(\hat{T},\hat{G}_t),T}$ and $f_{(\hat{T},\hat{G}_t),T-2}$ as accurately as possible so that their ratio in $\mathrm{P}_T(G^* = 0)$, and similarly in $\mathrm{P}_T(G^* = 1)$, is available. We will also need $\tau_{(\hat{T},\hat{G}_t)} = \hat{D}$ for the formula for $\mathrm{P}_T(G^* = 0)$.

Computing τ and σ

The equations describing the complex balance for the ODE reduce to the following system of two equations:

$$\kappa_1 \hat{M}^2 = \kappa_{-1}\hat{D}$$
$$\kappa_b \hat{D}\hat{G} = \kappa_u \hat{G}^*.$$

The second equation, together with $\hat{G}_t = \hat{G} + \hat{G}^*$, yields

$$\left(\hat{G}, \hat{G}^*\right) = \left(\frac{\kappa_u}{\kappa_b\hat{D} + \kappa_u}\,\hat{G}_t, \frac{\kappa_b\hat{D}}{\kappa_b\hat{D} + \kappa_u}\,\hat{G}_t\right). \tag{7}$$

The first equation becomes

$$\kappa_1(\hat{T} - 2\hat{D} - 2\hat{G}^*)^2 = \kappa_{-1}\hat{D},$$

and once we substitute the solution for $\hat{G}^*$ into the above and multiply both sides with $(\kappa_b\hat{D}+\kappa_u)^2$, we get a polynomial equation quartic in $\hat{D}$:

$$
\begin{aligned}
&4\kappa_1\kappa_b^2\hat{D}^4\\
&+(8\kappa_1\kappa_b^2\hat{G}_t-\kappa_{-1}\kappa_b^2+8\kappa_1\kappa_b\kappa_u-4\kappa_1\kappa_b^2\hat{T})\hat{D}^3\\
&+(4\kappa_1\kappa_b^2\hat{G}_t^2+8\kappa_1\kappa_b\kappa_u\hat{G}_t-2\kappa_{-1}\kappa_b\kappa_u+4\kappa_1\kappa_u^2-4\kappa_1\kappa_b^2\hat{G}_t\hat{T}-8\kappa_1\kappa_b\kappa_u\hat{T}+\kappa_1\kappa_b^2\hat{T}^2)\hat{D}^2\\
&+(-\kappa_{-1}\kappa_u^2-4\kappa_1\kappa_b\kappa_u\hat{G}_t\hat{T}-4\kappa_1\kappa_u^2\hat{T}+2\kappa_1\kappa_b\kappa_u\hat{T}^2)\hat{D}\\
&+\kappa_1\kappa_u^2\hat{T}^2=0.
\end{aligned}
$$

The calculations were partially carried out with the computer algebra system MATHEMATICA 8 (Wolfram Research, Inc.) and we justify some omissions in the text by these computational results. The MATHEMATICA notebooks are available in the electronic supplementary material.

We settled for the most straightforward choice for steady state, namely, the values of the stochastic model: $\hat{T}=T$ and $\hat{G}_t=1$. We suspect that with these two degrees of freedom there should be choices which result in a simpler calculation but we have not found one.

To avoid having to deal with a quartic equation, we leave $\hat{G}^*$ as an unknown parameter; since it is constrained to $[0,1]$, the uncertainty introduced is expected to be rather small. Note that this is true only if $\hat{G}_t$ is of order 1 in $\hat{T}$. Then the equation for $\hat{D}$ becomes

$$4\kappa_1\hat{D}^2+(8\kappa_1\hat{G}^*-\kappa_{-1}-4\kappa_1T)\hat{D}+4\kappa_1(\hat{G}^*)^2-4\kappa_1\hat{G}^*T+\kappa_1T^2=0.$$

The solution can be got by the quadratic formula. The unique solution is the smaller one, since the greater one is at least

$$-\frac{8\kappa_1\hat{G}^*-\kappa_{-1}-4\kappa_1T}{2\times4\kappa_1}=-\hat{G}^*+\frac{\kappa_{-1}}{8\kappa_1}+\frac{T}{2},$$

greater than $T/2-\hat{G}^*$, a natural upper bound of $\hat{D}$. The square root in the quadratic formula is approximated by Eq. (1), to yield

$$\tau_{(T,1)}=\hat{D}=\frac{T}{2}-c_\tau\sqrt{T}+c_\tau^2-\hat{G}^*+\left(c_\tau\hat{G}^*-\frac{c_\tau^3}{2}\right)\sqrt{\frac{1}{T}}+\mathcal{O}\left(T^{-3/2}\right),$$

$$\sigma_{(T,1)}=T-2\hat{D}-2\hat{G}^*=2c_\tau\sqrt{T}-2c_\tau^2+\left(c_\tau^3-2c_\tau\hat{G}^*\right)\sqrt{\frac{1}{T}}+\mathcal{O}\left(T^{-3/2}\right),$$

with $c_\tau=\sqrt{\frac{\kappa_{-1}}{8\kappa_1}}$. There is no limitation to how many terms these expansions can include but $\hat{G}^*$ is not specified beyond being confined to $[0,1]$.

It is interesting to note that $\hat{D}$, the steady state dimer count in the reaction rate equation, as $T\to\infty$, is essentially the maximum possible number of dimers, $T/2$, minus a correction whose order of magnitude is $\sqrt{T}$. This bias towards dimerisation versus dissociation reflects that dimerisation is described by a degree two intensity function, whereas dissociation by a linear intensity.

Computation of the coefficients by Cauchy's coefficient formula and the saddle point

To compute the coefficients $f_{(T,1),T}$ and $f_{(T,1),T-2}$ of $F_{(T,1)}(z)$ for evaluating $\mathrm{P}_T(G^* = 0)$, our guide will be the fine book [4]. The primary mission of analytical combinatorics is to assess the cardinality of sets of combinatorial structures of a certain size. This is achieved by taking the generating function whose nth coefficient is the cardinality of the class of the combinatorial structures of size n in question. This generating function is examined with complex analytical methods. Function

$$F_{(T,1)}(z) = \exp\left(\tau_{(T,1)} z^2 + \sigma_{(T,1)} z\right)$$

is an entire function, therefore its analysis will follow Chapter VIII, Saddle-point asymptotics. One of the case studies of this chapter is the analysis of function $z \mapsto \exp(\frac{1}{2}z^2 + z)$ (pp. 558–60), which our calculation will closely follow. It is perhaps interesting to note that $\exp(\tau z^2 + \sigma z)$ is the generating function of permutations with longest cycle at most two long (in other words, involutions) where 2τ marks inversions and σ marks fixed points [cf. 4, Ex. VIII.5, p. 558 and Ex. VIII.12, p. 569]. The topic of involutions was exploited in a study of dimerisation [6, Section 4].

The starting point is Cauchy's coefficient formula (2), with the integration curve γ a circle centred in the origin, which we transform into polar coordinates by substitution. For any $n \in \mathbb{N}$,

$$\begin{aligned} f_{(T,1),n} &= \frac{1}{2\pi i} \int_\gamma \frac{\exp\left(\tau_{(T,1)} z^2 + \sigma_{(T,1)} z\right)}{z^{n+1}} \, \mathrm{d}z \\ &= \frac{1}{2\pi i} \int_0^{2\pi} \frac{\exp\left(\tau_{(T,1)} r^2 e^{i\vartheta\, 2} + \sigma_{(T,1)} r e^{i\vartheta}\right)}{\left(re^{i\vartheta}\right)^{n+1}} \, re^{i\vartheta}\, i \, \mathrm{d}\vartheta \\ &= \frac{1}{2\pi} \int_0^{2\pi} \frac{\exp\left(\tau_{(T,1)} r^2 e^{i\,2\vartheta} + \sigma_{(T,1)} r e^{i\vartheta}\right)}{r^n e^{ni\vartheta}} \, \mathrm{d}\vartheta. \end{aligned} \tag{8}$$

In the integrand the division by z introduces a pole singularity at 0 to an otherwise entire function. The magnitude of the integrand tends to infinity as $z \to \pm\infty$ on the real axis because of the z^2 term. Therefore the magnitude of the integrand has two local minima on the real axis, which are additionally saddle points of the integrand. For illustration, see Figures 2 and 3. (Plots with the parameter values used in the *Numerical verification* are extremely similar to these and are not shown.)

The saddle point on the negative real halfline is exponentially small in T in comparison to the saddle point on the positive real half line. This is a corollary of the forthcoming Proposition 2: the positive saddle point dominates the origin-centred circle that passes through it; and the magnitude of the negative saddle point cannot be greater than the magnitude of the integrand at the intersection of the negative real halfline and the circle.

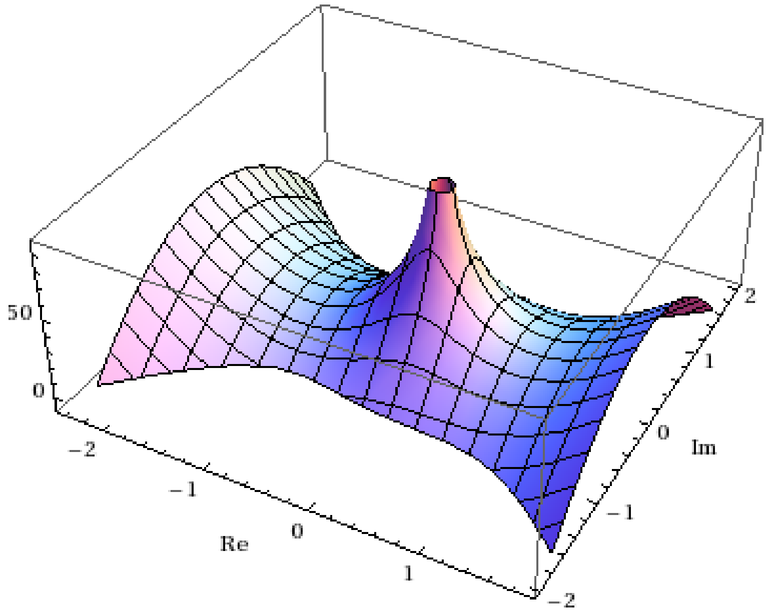

Figure 2: Base-10 log plot of $z \mapsto |\exp(\tau_{(100,1)}z^2 + \sigma_{(100,1)}z)/z^{101}|$ with parameters $\kappa_1 = 1$, $\kappa_{-1} = 6$, $\kappa_b = 0.1$, $\kappa_u = 2$, and an estimated $\hat{G}^* = 0.5$. On the vertical axis, 0 and 50 mark 10^0 and 10^{50}, respectively. The scales on the real and imaginary axes are linear.

The strategy suggested in [4] is to choose the integration curve such that it passes through (or near) the saddle point, in our case through the higher of the two saddle points. This technique is called the *saddle-point method*.

In order to find this higher saddle point, which is the saddle point on the positive real halfline, set $\vartheta = 0$, and define

$$\varphi_{(\hat{T},\hat{G}_t),n}(z) := \tau_{(\hat{T},\hat{G}_t)}z^2 + \sigma_{(\hat{T},\hat{G}_t)}z - n\log z.$$

The integral (8) becomes

$$f_{(T,1),n} = \frac{1}{2\pi}\int_{-\pi}^{\pi} \exp\left(\varphi_{(T,1),n}(re^{i\vartheta})\right)\,\mathrm{d}\vartheta.$$

The higher saddle point is the unique $r_{(\hat{T},\hat{G}_t),n} > 0$ that satisfies

$$\varphi'_{(\hat{T},\hat{G}_t),n}(r_{(\hat{T},\hat{G}_t),n}) = 0,$$

or

$$2\tau_{(\hat{T},\hat{G}_t)}r_{(\hat{T},\hat{G}_t),n} + \sigma_{(\hat{T},\hat{G}_t)} - \frac{n}{r_{(\hat{T},\hat{G}_t),n}} = 0. \tag{9}$$

For $(\hat{T},\hat{G}_t) = (T,1)$, the resulting quadratic equations have the positive roots

$$r_{(T,1),T-2} = 1 + (\hat{G}^* - 1)T^{-1} + c_\tau(\hat{G}^* - 1)T^{-3/2} + \mathcal{O}\left(T^{-2}\right),$$
$$r_{(T,1),T} = 1 + \hat{G}^*T^{-1} + c_\tau\hat{G}^*T^{-3/2} + \mathcal{O}\left(T^{-2}\right).$$

These are the radii to be used in Cauchy's coefficient formula.

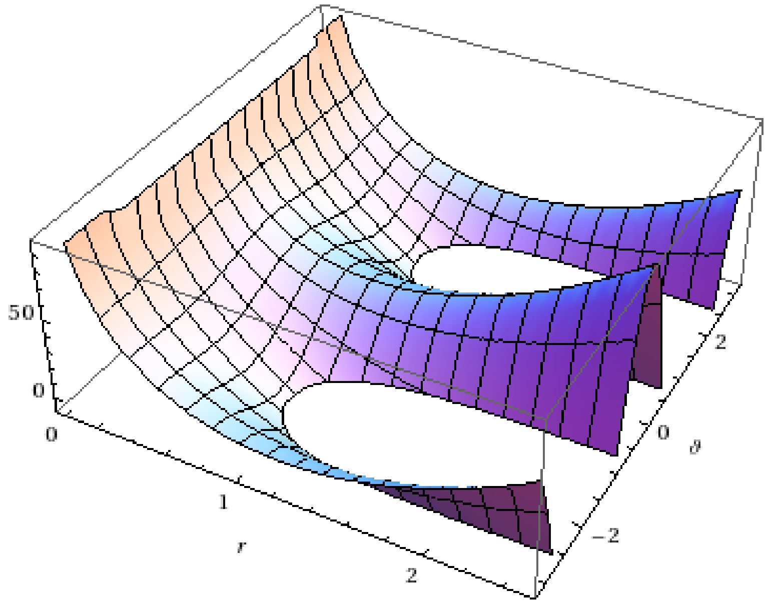

Figure 3: Base-10 log plot of $(r,\vartheta) \mapsto |\exp(\tau_{(100,1)}r^2e^{i\,2\vartheta} + \sigma_{(100,1)}re^{i\vartheta})/(r^{100}e^{100i\vartheta})|$ with parameters $\kappa_1 = 1$, $\kappa_{-1} = 6$, $\kappa_b = 0.1$, $\kappa_u = 2$, and an estimated $\hat{G}^* = 0.5$. On the vertical axis, 0 and 50 mark 10^0 and 10^{50}, respectively. The scales on the r and ϑ axes are linear.

Decomposition of the integral

Consider the following formulation of the integral,

$$f_{(T,1),n} = \frac{\exp\left(\varphi_{(T,1),n}(r)\right)}{2\pi} \int_{-\pi}^{\pi} \exp\left(\varphi_{(T,1),n}(re^{i\vartheta}) - \varphi_{(T,1),n}(r)\right) \mathrm{d}\vartheta,$$

and the resulting definition

$$I_{(T,1),n}(r) := \int_{-\pi}^{\pi} \exp\left(\varphi_{(T,1),n}(re^{i\vartheta}) - \varphi_{(T,1),n}(r)\right) \mathrm{d}\vartheta.$$

For $\mathrm{P}_T(G^* = 0)$, we factorise $f_{(T,1),T-2}/f_{(T,1),T}$ according to this decomposition as

$$\frac{f_{(T,1),T-2}}{f_{(T,1),T}} = \frac{\exp\left(\varphi_{(T,1),T-2}(r_{(T,1),T-2})\right)}{\exp\left(\varphi_{(T,1),T}(r_{(T,1),T})\right)} \frac{I_{(T,1),T-2}(r_{(T,1),T-2})}{I_{(T,1),T}(r_{(T,1),T})}. \tag{10}$$

In the first factor, by the definition of $\varphi_{(T,1),n}(z)$ and the results for $\sigma_{(T,1)}$, $\tau_{(T,1)}$, $r_{(T,1),T-2}$, and $r_{(T,1),T}$, we have

$$\begin{aligned}
&\varphi_{(T,1),T-2}(r_{(T,1),T-2}) - \varphi_{(T,1),T}(r_{(T,1),T}) = \\
&\qquad = \tau_{(T,1)}r^2_{(T,1),T-2} + \sigma_{(T,1)}r_{(T,1),T-2} - (T-2)\log r_{(T,1),T-2} \\
&\qquad\quad - \left(\tau_{(T,1)}r^2_{(T,1),T} + \sigma_{(T,1)}r_{(T,1),T} - T\log r_{(T,1),T}\right) \\
&\qquad = (2\hat{G}^* - 1)T^{-1} + c_\tau(2\hat{G}^* - 1)T^{-3/2} + \mathcal{O}(T^{-2}),
\end{aligned}$$

and after exponentiation

$$\frac{\exp\left(\varphi_{(T,1),T-2}(r_{(T,1),T-2})\right)}{\exp\left(\varphi_{(T,1),T}(r_{(T,1),T})\right)} = 1 + (2\hat{G}^* - 1)T^{-1} + c_\tau(2\hat{G}^* - 1)T^{-3/2} + \mathcal{O}(T^{-2}). \quad (11)$$

Computation of the integral

The integral $I_{(T,1),n}(r_{(T,1),n})$ will be evaluated separately on a central part and on an outside part:

$$\begin{aligned} I_{(T,1),n}(r) &= \int_{-\pi}^{\pi} \exp\Big(\varphi_{(T,1),n}(re^{i\vartheta}) - \varphi_{(T,1),n}(r)\Big)\,\mathrm{d}\vartheta \\ &= \int_{-\theta_0}^{\theta_0} \exp\Big(\varphi_{(T,1),n}(re^{i\vartheta}) - \varphi_{(T,1),n}(r)\Big)\,\mathrm{d}\vartheta \\ &\quad + \int_{\theta_0}^{2\pi-\theta_0} \exp\Big(\varphi_{(T,1),n}(re^{i\vartheta}) - \varphi_{(T,1),n}(r)\Big)\,\mathrm{d}\vartheta. \end{aligned}$$

The integral outside the central part will be shown to be negligibly small (Proposition 2). This quantity will be the first error term, ε_1. This is a consequence of the integrand being very small on the integration path far from the saddle point.

The central integral will be approximated with a Gaussian integral. This approximation will give two error terms. On the one hand, ε_2 will reflect the difference between the genuine central integral and its Gaussian approximation (Proposition 3). On the other hand, ε_3 is the error from falsely computing the tails in a complete Gaussian integral when only the incomplete integral on the central part is needed (Proposition 4).

The boundary θ_0 between the central and the non-central parts is chosen according to the principles in [4, Eq. (21), p. 554], and fixed to be $\theta_0 = n^{-2/5}$.

Proposition 2 There exists a constant $c \in]0,1[$ such that

$$\begin{aligned} \int_{\theta_0}^{2\pi-\theta_0} \exp\Big(\varphi_{(T,1),T-2}(r_{(T,1),T-2}e^{i\vartheta}) - \varphi_{(T,1),T-2}(r_{(T,1),T-2})\Big)\,\mathrm{d}\vartheta &= \mathcal{O}(e^{-cT^{1/5}}), \\ \int_{\theta_0}^{2\pi-\theta_0} \exp\Big(\varphi_{(T,1),T}(r_{(T,1),T}e^{i\vartheta}) - \varphi_{(T,1),T}(r_{(T,1),T})\Big)\,\mathrm{d}\vartheta &= \mathcal{O}(e^{-cT^{1/5}}). \end{aligned}$$

Proof The claim will be proved if the integrands are bounded from above by $\mathcal{O}(e^{-cT^{1/5}})$ since the interval of integration is no longer than 2π. The exponent will be rewritten in a different form. For brevity, let $\sigma := \sigma_{(T,1)}$, $\tau := \tau_{(T,1)}$ and $r_n := r_{(T,1),n}$.

$$\begin{aligned} &\varphi_{(T,1),n}(r_{(T,1),n}e^{i\vartheta}) - \varphi_{(T,1),n}(r_{(T,1),n}) = \\ &\qquad = \tau r_n^2 e^{i\,2\vartheta} + \sigma r_n e^{i\vartheta} - n\log(r_n e^{i\vartheta}) - (\tau r_n^2 + \sigma r_n - n\log r_n) \\ &\qquad = \tau r_n^2(e^{i\,2\vartheta} - 1) + \sigma r_n(e^{i\vartheta} - 1) - n(i\vartheta). \end{aligned}$$

r_n was chosen in such a way that $\varphi'_{(T,1),n}(r_n) = 0$, that is,

$$2\tau r_n + \sigma - \frac{n}{r_n} = 0.$$

From this, $-n = -2\tau r_n^2 - \sigma r_n$, and

$$\varphi_{(T,1),n}(r_{(T,1),n}e^{i\vartheta}) - \varphi_{(T,1),n}(r_{(T,1),n}) = \\ = \tau r_n^2(e^{i\,2\vartheta} - 1 - 2(i\vartheta)) + \sigma r_n(e^{i\vartheta} - 1 - i\vartheta).$$

The magnitude of an exponentiated term is the exponential of its real part, thus

$$\left|\exp\left(\varphi_{(T,1),n}(r_{(T,1),n}e^{i\vartheta}) - \varphi_{(T,1),n}(r_{(T,1),n})\right)\right| = \\ = \exp\left(\tau r_n^2(\cos(2\vartheta) - 1) + \sigma r_n(\cos\vartheta - 1)\right).$$

Consider the function $g:\ \vartheta \mapsto \tau r_n^2(\cos(2\vartheta) - 1) + \sigma r_n(\cos\vartheta - 1)$. First, $g(0) = 0$. On $[0, \frac{\pi}{2}]$, both $\cos(2\vartheta)$ and $\cos\vartheta$ are decreasing, and so is g. On $[\frac{\pi}{2}, \frac{3\pi}{4}]$, both $\cos(2\vartheta)$ and $\cos\vartheta$ are nonpositive, and $g(\vartheta) \le -\tau r_n^2 - \sigma r_n$. Finally, on $[\frac{3\pi}{4}, \pi]$, $\cos\vartheta \le -\frac{\sqrt{2}}{2}$ and $g(\vartheta) \le -\frac{\sqrt{2}+2}{2}\sigma r_n$. The same argument can be used for $[-\pi, 0]$.

Using Eq. (3), the above becomes

$$\left|\exp\left(\varphi_{(T,1),n}(r_{(T,1),n}e^{i\vartheta}) - \varphi_{(T,1),n}(r_{(T,1),n})\right)\right| = \\ = \exp\left(\tau r_n^2\left(2\vartheta^2 + \mathcal{O}(\vartheta^4)\right) + \sigma r_n\left(\frac{\vartheta^2}{2} + \mathcal{O}(\vartheta^4)\right)\right).$$

These considerations give for $n \in \{T-2, T\}$ and $\theta_0 = n^{-2/5}$ that $e^{g(\vartheta)}$ is exponentially small outside of $[-\theta_0, \theta_0]$: on $[\theta_0, \frac{\pi}{2}] \cup [-\frac{\pi}{2}, -\theta_0]$,

$$\left|\exp\left(\varphi_{(T,1),n}(r_{(T,1),n}e^{i\vartheta}) - \varphi_{(T,1),n}(r_{(T,1),n})\right)\right| \le \\ \left|\exp\left(\varphi_{(T,1),n}(r_{(T,1),n}e^{i\theta_0}) - \varphi_{(T,1),n}(r_{(T,1),n})\right)\right| = \\ = \exp\left(-T^{1/5} + c_\tau T^{-3/10} + \mathcal{O}(T^{-3/5})\right)$$

is certainly an upper bound (for the last equality, see the MATHEMATICA notebook), while on $[\frac{\pi}{2}, \frac{3\pi}{2}]$, $g(\vartheta) \le -\sigma r_n$ gives an upper bound $e^{-2c_\tau T^{1/2} + \mathcal{O}(1)}$. Therefore, the first error term for both $n = T-2$ and $n = T$ is $\varepsilon_1 = \mathcal{O}(e^{-cT^{1/5}})$. □

Proposition 3 For $n \in \{T-2, T\}$,

$$\int_{-\theta_0}^{\theta_0} \exp\left(\varphi_{(T,1),n}(r_{(T,1),n}e^{i\vartheta}) - \varphi_{(T,1),n}(r_{(T,1),n})\right) \mathrm{d}\vartheta = \\ = \int_{-\theta_0}^{\theta_0} \exp\left(-\left(n - \frac{\sigma_{(T,1)}}{2} r_{(T,1),n}\right)\vartheta^2\right) \mathrm{d}\vartheta\left(1 + \mathcal{O}(T^{-1/5})\right).$$

Proof To evaluate the central integral

$$\int_{-\theta_0}^{\theta_0} \exp\left(\varphi_{(T,1),n}(r_{(T,1),n}e^{i\vartheta}) - \varphi_{(T,1),n}(r_{(T,1),n})\right) \mathrm{d}\vartheta,$$

a Taylor expansion of $\varphi_{(T,1),n}$ will be used. For $\vartheta \in [-\theta_0, \theta_0]$,

$$\begin{aligned}
&\varphi_{(T,1),n}(r_{(T,1),n}e^{i\vartheta}) - \varphi_{(T,1),n}(r_{(T,1),n}) = \\
&\qquad = \frac{\varphi'_{(T,1),n}(r_{(T,1),n})}{1} r_{(T,1),n}(e^{i\vartheta}-1) + \frac{\varphi''_{(T,1),n}(r_{(T,1),n})}{2} r^2_{(T,1),n}(e^{i\vartheta}-1)^2 \\
&\qquad + \frac{\varphi'''_{(T,1),n}(r_{(T,1),n})}{6} r^3_{(T,1),n}(e^{i\vartheta}-1)^3 + \mathcal{O}(\vartheta^4).
\end{aligned}$$

Recall that $r_{(T,1),n}$ is chosen in such a way that $\varphi'_{(T,1),n}(r_{(T,1),n}) = 0$. Further, from Eq. (9),

$$\begin{aligned}
\varphi''_{(T,1),n}(r_{(T,1),n})r^2_{(T,1),n} &= 2\tau_{(T,1)}r^2_{(T,1),n} + n = 2n - \sigma_{(T,1)}r_{(T,1),n}, \\
\varphi'''_{(T,1),n}(r_{(T,1),n})r^3_{(T,1),n} &= -2n.
\end{aligned}$$

From

$$e^{i\vartheta} = 1 + i\vartheta - \frac{1}{2}\vartheta^2 - \frac{i}{6}\vartheta^3 + \mathcal{O}(\vartheta^4),$$

it follows

$$\begin{aligned}
(e^{i\vartheta}-1)^2 &= -\vartheta^2 - i\vartheta^3 + \mathcal{O}(\vartheta^4), \\
(e^{i\vartheta}-1)^3 &= -i\vartheta^3 + \mathcal{O}(\vartheta^4).
\end{aligned}$$

The exponent of the integrand reduces to

$$\begin{aligned}
&\varphi_{(T,1),n}(r_{(T,1),n}e^{i\vartheta}) - \varphi_{(T,1),n}(r_{(T,1),n}) = \\
&\qquad = \left(n - \frac{\sigma_{(T,1)}}{2} r_{(T,1),n}\right)(e^{i\vartheta}-1)^2 - \frac{n}{3}(e^{i\vartheta}-1)^3 + \mathcal{O}(\vartheta^4),
\end{aligned}$$

where the constraints $\vartheta \in [-\theta_0, \theta_0] = [-n^{-2/5}, n^{-2/5}]$ and $n \in \{T-2, T\}$ allow further simplification:

$$\begin{aligned}
&\varphi_{(T,1),n}(r_{(T,1),n}e^{i\vartheta}) - \varphi_{(T,1),n}(r_{(T,1),n}) = \\
&\qquad = -\left(n - \frac{\sigma_{(T,1)}}{2} r_{(T,1),n}\right)\vartheta^2 + \mathcal{O}(T^{-1/5}).
\end{aligned}$$

After exponentiation, $\exp(\mathcal{O}(T^{-1/5})) = 1 + \mathcal{O}(T^{-1/5})$. Let this error term minus one be denoted by ε_2, so that $\varepsilon_2 = \mathcal{O}(T^{-1/5})$. Ultimately, for $n \in \{T-2, T\}$, the central integral is approximated by a Gaussian integral,

$$\begin{aligned}
&\int_{-\theta_0}^{\theta_0} \exp\Big(\varphi_{(T,1),n}(r_{(T,1),n}e^{i\vartheta}) - \varphi_{(T,1),n}(r_{(T,1),n})\Big)\,\mathrm{d}\vartheta = \\
&\qquad = \int_{-\theta_0}^{\theta_0} \exp\left(-\left(n - \frac{\sigma_{(T,1)}}{2} r_{(T,1),n}\right)\vartheta^2\right)\mathrm{d}\vartheta\Big(1 + \mathcal{O}(T^{-1/5})\Big). \qquad \square
\end{aligned}$$

In the next calculation, the central, approximating Gaussian integral will be evaluated.

Proposition 4 There exists a constant $c \in]0,1[$ such that for $n \in \{T-2,\, T\}$,

$$\int_{-n^{-2/5}}^{n^{-2/5}} \exp\left(-\left(n - \frac{\sigma_{(T,1)}}{2} r_{(T,1),n} \right) \vartheta^2 \right) \mathrm{d}\vartheta = $$
$$= \sqrt{\frac{\pi}{n - \frac{\sigma_{(T,1)}}{2} r_{(T,1),n}}} + \mathcal{O}\left(e^{-cT^{1/5}} \right).$$

Proof In the following calculation, we first integrate by substitution. In the second step, we complete the Gaussian integral and simultaneously subtract the two tail integrals. The third step follows from Eq. (4) and we use that the tails of the normal distribution are exponentially small, Eq. (5).

$$\int_{-n^{-2/5}}^{n^{-2/5}} \exp\left(-\left(n - \frac{\sigma_{(T,1)}}{2} r_{(T,1),n} \right) \vartheta^2 \right) \mathrm{d}\vartheta =$$
$$= \frac{1}{\sqrt{2\left(n - \frac{\sigma_{(T,1)}}{2} r_{(T,1),n}\right)}} \int_{-\sqrt{2\left(n-\frac{\sigma_{(T,1)}}{2} r_{(T,1),n}\right)}\, n^{-2/5}}^{\sqrt{2\left(n-\frac{\sigma_{(T,1)}}{2} r_{(T,1),n}\right)}\, n^{-2/5}} \exp\left(-\frac{\varrho^2}{2} \right) \mathrm{d}\varrho$$
$$= \frac{1}{\sqrt{2\left(n - \frac{\sigma_{(T,1)}}{2} r_{(T,1),n}\right)}} \int_{-\infty}^{\infty} \exp\left(-\frac{\varrho^2}{2} \right) \mathrm{d}\varrho$$
$$- \frac{2}{\sqrt{2\left(n - \frac{\sigma_{(T,1)}}{2} r_{(T,1),n}\right)}} \int_{\sqrt{2\left(n-\frac{\sigma_{(T,1)}}{2} r_{(T,1),n}\right)}\, n^{-2/5}}^{\infty} \exp\left(-\frac{\varrho^2}{2} \right) \mathrm{d}\varrho$$
$$= \frac{1}{\sqrt{2\left(n - \frac{\sigma_{(T,1)}}{2} r_{(T,1),n}\right)}} \sqrt{2\pi}$$
$$+ \frac{2}{\sqrt{2\left(n - \frac{\sigma_{(T,1)}}{2} r_{(T,1),n}\right)}} \mathcal{O}\left(\exp\left(-\frac{1}{2}\, 2\left(n - \frac{\sigma_{(T,1)}}{2} r_{(T,1),n} \right) n^{-4/5} \right) \right)$$
$$= \sqrt{\frac{\pi}{n - \frac{\sigma_{(T,1)}}{2} r_{(T,1),n}}} + \frac{2}{\sqrt{2\left(n - \frac{\sigma_{(T,1)}}{2} r_{(T,1),n}\right)}} \mathcal{O}\left(e^{-cT^{1/5}} \right)$$
$$= \sqrt{\frac{\pi}{n - \frac{\sigma_{(T,1)}}{2} r_{(T,1),n}}} + \mathcal{O}\left(e^{-cT^{1/5}} \right).$$

Let ε_3 denote this last error term. □

As a corollary of Propositions 2–4, we get for $n \in \{T-2,\, T\}$,

$$I_{(T,1),n}(r_{(T,1),n}) = \left(\sqrt{\frac{\pi}{n - \frac{\sigma_{(T,1)}}{2} r_{(T,1),n}}} + \varepsilon_3 \right) (1 + \varepsilon_2) + \varepsilon_1.$$

Consequently,

$$\frac{I_{(T,1),T-2}(r_{(T,1),T-2})}{I_{(T,1),T}(r_{(T,1),T})} = 1 + \mathcal{O}(T^{-1/5}).$$

Together with Eq. (11), from Eq. (10), this gives

$$\frac{f_{(T,1),T-2}}{f_{(T,1),T}} = 1 + \mathcal{O}(T^{-1/5}). \tag{12}$$

By substituting this into Eq. (6), we get the claim of the main text.

It is noteworthy that ultimately Eq. (6) is revealed to be almost identical to its deterministic counterpart, Eq. (7), the difference being the error term. This can be seen as the exchanging of limits: whether to first take the large volume limit with one gene and then take time to infinity (deterministic steady state) or to take time to infinity first and then increase the number of transcription factors (equilibrium distribution) has been shown to be equivalent modulo the error term. It is somewhat puzzling that such effort was needed to compute by Cauchy's coefficient formula two coefficients independently, which eventually turned out to be almost equal, Eq. (12). We wonder if there is a trick or a formula to directly compute the ratio of two coefficients of a power series.

Analogously to Eq. (6), $\mathrm{P}_T(G^* = 1)$ can be expressed as

$$\mathrm{P}_T(G^* = 1) = \frac{\kappa_b \hat{D}}{\kappa_b \hat{D} + \kappa_u \frac{[z^T] \exp\left(\hat{D}z^2 + (\hat{T} - 2\hat{D} - 2\hat{G}^*)z\right)}{[z^{T-2}] \exp\left(\hat{D}z^2 + (\hat{T} - 2\hat{D} - 2\hat{G}^*)z\right)}}.$$

After substituting $\hat{D} = \tau_{(T,1)}$ and $f_{(T,1),T}/f_{(T,1),T-2} = 1 + \mathcal{O}(T^{-1/5})$, one arrives at

$$\begin{aligned}
\mathrm{P}_T(G^* = 1) &= \kappa_b \left(\frac{T}{2} - \sqrt{\frac{\kappa_{-1}}{8\kappa_1}} T^{1/2} + \frac{\kappa_{-1}}{8\kappa_1} - \hat{G}^* + \mathcal{O}(T^{-1/2}) \right) \times \\
&\quad \times \left(\kappa_b \left(\frac{T}{2} - \sqrt{\frac{\kappa_{-1}}{8\kappa_1}} T^{1/2} + \frac{\kappa_{-1}}{8\kappa_1} + \frac{\kappa_u}{\kappa_b} - \hat{G}^* + \mathcal{O}(T^{-1/5}) \right) \right)^{-1} \\
&= 1 - \frac{2\kappa_u}{\kappa_b} T^{-1} - \frac{4\kappa_u}{\kappa_b} \sqrt{\frac{\kappa_{-1}}{8\kappa_1}} T^{-3/2} + 4 \frac{\kappa_u}{\kappa_b} \left(\frac{\kappa_u}{\kappa_b} - \frac{\kappa_{-1}}{8\kappa_1} \right) T^{-2} + \mathcal{O}(T^{-6/5}).
\end{aligned} \tag{13}$$

In the last expression, terms that are smaller than the error term are also given, allowing for the possibility that the approximation via the saddle-point method is more accurate than what error term we could prove. That this might really be the case is suggested by the higher accuracy of Eq. (13) in comparison to the formula of the main text for $\mathrm{P}_T(G^* = 0)$ in the forthcoming numerical verification (Table 1 and Figure 4). Note, however, that the numerical verification is limited to only one set of parameter values.

Numerical verification

In order to verify the calculation, the result was checked against numerical simulation by Gillespie's stochastic simulation algorithm [1]. The simulations were conducted in

GNU OCTAVE with code that is compatible with MATLAB (The MathWorks, Inc.) also. Parameter values were chosen in the ranges considered biologically realistic by [7, Supporting Material]. These are TF dimerisation rate $\kappa'_1 = 5 \times 10^7$ $\mathrm{M}^{-1}\mathrm{s}^{-1}$, TF dimer dissociation rate $\kappa'_{-1} = 1$ s^{-1}, gene–TF binding rate $\kappa'_b = 10^8$ $\mathrm{M}^{-1}\mathrm{s}^{-1}$, and gene–TF complex dissociation rate $\kappa'_u = 5$ s^{-1}. We take the cell volume to be $V = 10^{-15}$ ℓ and the Avogadro constant $N_A = 6 \times 10^{23}$ mol^{-1}. The rate constants are converted from the continuous (concentration) formalism into the discrete (count) formalism:

$$\begin{aligned}
\kappa_1 &= \kappa'_1/(N_A V) = 8.33\ldots \times 10^{-2}\ \mathrm{s}^{-1},\\
\kappa_{-1} &= \kappa'_{-1} = 1\ \mathrm{s}^{-1},\\
\kappa_b &= \kappa'_b/(N_A V) = 0.166\ldots\ \mathrm{s}^{-1},\\
\kappa_u &= \kappa'_u = 5\ \mathrm{s}^{-1}.
\end{aligned}$$

Each empirical probability value resulted from 3000 samples. Sampling took place at the end of independent trajectories of 60 s length from initial state $(M, D, G, G^*) = (T, 0, 1, 0)$. Based on the observation of individual runs with the naked eye, we are confident that for practical purposes, equilibrium distribution was comfortably reached by this time.

Estimates by the two formulae are computed by evaluating them without the error terms. For $1 - \mathrm{P}_T(G^* = 0)$, the formula of the main text is used. For $\mathrm{P}_T(G^* = 1)$, Eq. (13) is used. Its result is an interval since the value $\hat{G}^*$ is only known to lie in $[0, 1]$. For instance, for the lower bound, $\hat{G}^*$ is set to 1 in the enumerator and to 0 in the denominator.

For increasing values of T, the estimates for $\mathrm{P}_T(G^* = 1)$ are indeed approaching the simulated value (Table 1 and Figure 4). One can observe that the formulae can predict probabilities outside of $[0, 1]$ but the problem is naturally solved as T increases. Due to differences in the truncation of error terms, $1 - \mathrm{P}_T(G^* = 0)$ is not necessarily in the interval given by Eq. (13).

Even as T tends to infinity, owing to sampling error, one cannot expect a perfect match between simulation results and the analytic estimates. If we knew that $\mathrm{P}_T(G^* = 1)$ is exactly $p = (0.82, 0.94, 0.98)$, then the standard deviation of the numerical estimates would be $\sqrt{p(1-p)/3000} \approx (0.0070, 0.0043, 0.0026)$, respectively. Our simulation results are consistent with these expected sampling errors. Generating the 3000 samples took about 6.5 h with $T = 1000$ and about 108 h with $T = 3162$ on the computer described in *Connecting fast and slow time scales: A demonstration.*

References

[1] Gillespie DT. Exact stochastic simulation of coupled chemical reactions. The Journal of Physical Chemistry. 1977;81(25):2340–2361. doi:10.1021/j100540a008.

T	Simulation	Estimate by $1 - \mathrm{P}_T(G^* = 0)$	Estimate by (13)
10	0.0683	-5.0000	[0.0499, 0.0831]
32	0.2627	-0.8750	[0.2359, 0.2671]
100	0.5777	0.4000	[0.5524, 0.5751]
316	0.8277	0.8101	[0.8152, 0.8261]
1000	0.9353	0.9400	[0.9371, 0.9410]
3162	0.9797	0.9810	[0.9799, 0.9812]

Table 1: Comparison of $\mathrm{P}_T(G^* = 1)$ values from numerical simulation and from the two analytical formulae.

[2] Adan I, Resing J. Circular Markov chains. Eindhoven, The Netherlands: Eindhoven University of Technology; 1996. Memorandum COSOR 96-16. Available from: http://alexandria.tue.nl/repository/books/461817.pdf.

[3] Abramowitz M, Stegun IA, editors. Handbook of mathematical functions with formulas, graphs, and mathematical tables. Applied Mathematics Series - 55. Washington, D.C., USA: U.S. Government Printing Office; 1972. Available from: http://www.nr.com/aands/.

[4] Flajolet P, Sedgewick R. Analytic combinatorics. Cambridge, UK: Cambridge University Press; 2009. E-version. Available from: http://algo.inria.fr/flajolet/Publications/book.pdf.

[5] Anderson DF, Craciun G, Kurtz TG. Product-form stationary distributions for deficiency zero chemical reaction networks. Bulletin of Mathematical Biology. 2010;72:1947–1970. doi:10.1007/s11538-010-9517-4.

[6] Fournier T, Gabriel JP, Mazza C, Pasquier J, Galbete J, Mermod N. Stochastic models and numerical algorithms for a class of regulatory gene networks. Bulletin of Mathematical Biology. 2009;71(6):1394–1431. Available from: http://dx.doi.org/10.1007/s11538-009-9407-9. doi:10.1007/s11538-009-9407-9.

[7] Marquez-Lago TT, Stelling J. Counter-intuitive stochastic behavior of simple gene circuits with negative feedback. Biophysical Journal. 2010;98(9):1742–1750. Available from: http://www.sciencedirect.com/science/article/pii/S0006349510001499. doi:10.1016/j.bpj.2010.01.018.

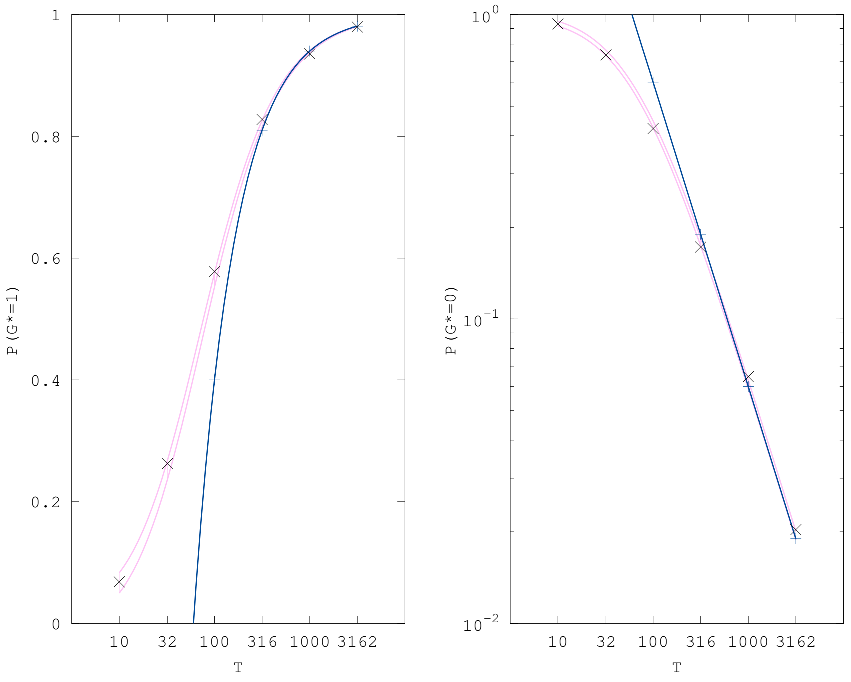

Figure 4: Plot of data in Table 1. Simulation results are marked by black X symbols, the estimates by the formula for $1 - \mathrm{P}_T(G^* = 0)$ by blue + symbols and a connecting line, and the interval estimates by the formula (13) for $\mathrm{P}_T(G^* = 1)$ by pink vertical bars and connecting lines. The right panel uses a logarithmic scale on the y-axis to magnify the approach to 1.
